# The dopamine hypothesis for ADHD: An evaluation of evidence accumulated from human studies and animal models

**DOI:** 10.3389/fpsyt.2024.1492126

**Published:** 2024-11-15

**Authors:** Hayley J. MacDonald, Rune Kleppe, Peter D. Szigetvari, Jan Haavik

**Affiliations:** ^1^ Department of Biological and Medical Psychology, University of Bergen, Bergen, Norway; ^2^ Norwegian Centre for Maritime- and Diving Medicine, Department of Occupational Medicine, Haukeland University Hospital, Bergen, Norway; ^3^ Department of Biomedicine, University of Bergen, Bergen, Norway; ^4^ Division of Psychiatry, Haukeland University Hospital, Bergen, Norway

**Keywords:** dopamine, monoamines, attention deficit/hyperactivity disorder, stimulants, genetics, neurometabolic disorders, psychopharmacology, neuropsychological disorders

## Abstract

Multiple lines of evidence indicate that altered dopamine signaling may be involved in neuropsychiatric disorders and common behavioral traits. Here we critically review evidence collected during the past 40-plus years supporting the role of dopamine dysfunction in attention deficit hyperactivity disorder (ADHD). We recapitulate the basic components of dopaminergic signaling in the central nervous system, focusing on core enzymes, transporters and receptors involved in monoaminergic functions, particularly in striatal and cortical regions. We summarize key human brain imaging and genetic studies reporting associations between dopaminergic neurotransmission and behavioral traits, with an emphasis on ADHD. We also consider ADHD in the context of animal models and single gene, metabolic, and neurological disorders with established dysfunction of the dopaminergic system. Examining the evidence in this way leads us to conclude that there is evidence for the involvement of dopamine but limited evidence for a hypo-dopaminergic state *per se* as a key component of ADHD. We propose a path forward to increase our understanding of dopamine signaling in human behavioral traits and disorders that should particularly focus on its role in clinical subgroups, during brain development and how it interacts with other neurotransmitter systems.

## Introduction

Dopamine (DA) was synthesized in 1910 (1) and during the 1950s it was reported to be present in brain tissue (2). Following the discoveries that DA is a neurotransmitter (3) and that DA receptors are drug targets (3), the role of the DA system has been explored in many neuropsychiatric disorders. In particular, the observation of a reduced DA content in postmortem brain tissue from Parkinson’s disease patients (4) spurred an intense interest in the role of DA in neurological and psychiatric disorders and other conditions. However, in comparison to the well-established role of DA deficiency in neurodegenerative diseases of the basal ganglia and rare neurometabolic syndromes affecting DA synthesis, transport or metabolism (5), research on dopamine’s role in most other traits has been challenging and subject to controversy.

### Evidence linking the dopamine system and attention deficit hyperactivity disorder

Attention deficit hyperactivity disorder (ADHD) is characterized by dominant symptoms of inattention (e.g. being sidetracked by external or unimportant stimuli) and/or hyperactivity (e.g. squirming or fidgeting while seated) and impulsiveness (e.g. difficulty waiting your turn). According to the current version of the Diagnostic and Statistical Manual for Mental Disorders (DSM-5), these symptoms need to persist for longer than six months, be observed in at least two settings, and negatively impact academic/social/occupational functioning to qualify for a diagnosis of ADHD (in addition to other diagnostic criteria) (6). It has long been speculated that ADHD symptoms may be related to some alterations in monoaminergic and in particular dopaminergic neurotransmission (7, 8). While it has been difficult to pinpoint exactly how a postulated dysfunction of DA occurs in ADHD, this hypothesis has gained much support in the scientific community and not the least in popular media (9). According to some “influencers”, popular books and other media intended for the lay audience, ADHD patients lack DA in their brain (10). Despite this widely held popular opinion, the scientific evidence supporting this version of the DA hypothesis is not necessarily present. Indeed, a simplistic portrayal of ADHD as a general DA deficiency syndrome might be one of the most common misconceptions regarding ADHD neurobiology. This is not the only misconception about ADHD that is widely distributed in social media. In a quantitative study of social media content quality, it was found that 52% of the 100 most popular videos on ADHD on TikTok were classified as misleading (11).

Scientifically, the DA hypothesis of ADHD has been based on several lines of evidence, including: 1) stimulant drugs target the monoamine systems, including DA, 2) animal models genetically engineered for altered DA homeostasis mimic some symptoms of ADHD, 3) brain imaging studies implicate the DA system, and 4) studies of genetic variants in the DA system in humans (9). However, ever since the DA hypothesis was first proposed about 40 years ago some researchers have also been skeptical of it. For example, when focusing on the effect of medications used for ADHD, the 1987 review by Zametkin and Rapoport (12) found evidence against the DA hypothesis when considering the limited effects of DA agonists and antagonists. They instead reported evidence primarily favoring the noradrenergic system.

**Figure 1:**
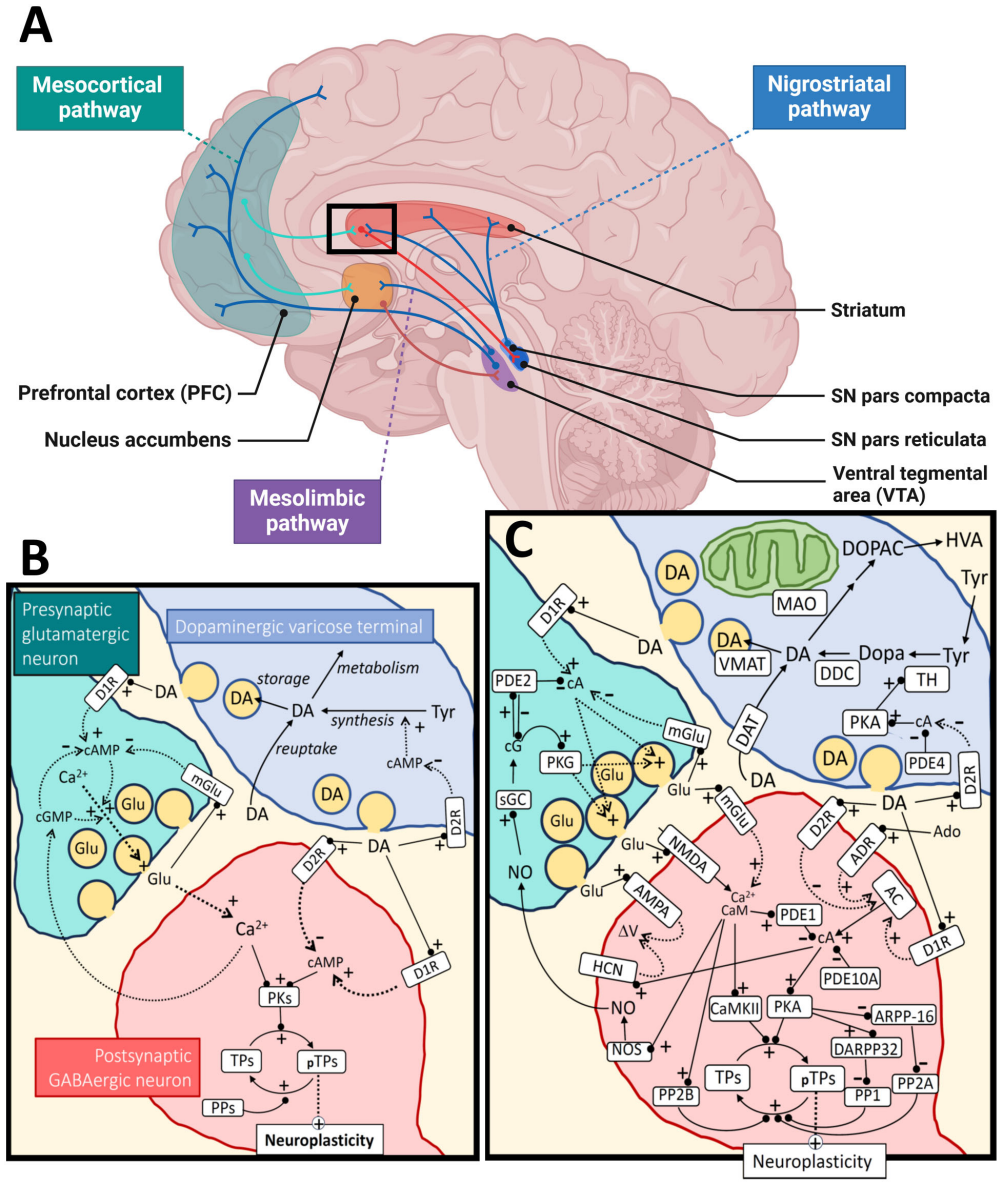
Dopamine metabolism and signaling. Panel **(A)** shows the major dopaminergic pathways in the human brain originating from the *substantia nigra* (*SN*) *pars compacta* for the nigrostriatal pathway and the ventral tegmental area (VTA) for the mesolimbic and mesocortical pathways. For simplicity, the panel shows only one of the output pathways (the direct pathway) from the striatal medium spiny neurons (of the D1R-type) to the *SN pars reticulata* (or globus pallidus internal). The modulation of cortical input to the striatum by dopamine (black square) is further illustrated at the synaptic and molecular level in panel **(B)**, as a simplification, and in more detail in panel **(C)**. The regulation of neuroplasticity by dopamine is shown for a cortico-striatal synapse under influence by a nearby dopaminergic varicose release site. Some of the relevant cyclic nucleotides [cAMP (cA), cGMP (cG)] and calcium signal interactions are shown for both the presynaptic cortical glutamatergic neuron and a postsynaptic GABAergic medium spiny neuron. For the dopaminergic terminal (upper right), synthesis and metabolism of dopamine are shown as well as a simplified regulation of TH activity through cAMP/PKA signaling, which is inhibited by D2 autoreceptors. The change in neuroplasticity is mediated by different target proteins (TPs; pTPs – phosphorylated TPs) of cAMP-dependent protein kinase (PKA) and calcium/calmodulin dependent protein kinase II (CaMKII). Examples of such TPs are CREB (cAMP-response element binding), STEP (striatal-enriched protein tyrosine phosphatase), proteins involved in cytoskeletal restructuring, AMPA receptors, etc. AMPA, α-amino-3-hydroxy-5-methyl-4-isoxazolepropionic acid receptor; AC, adenylate cyclase; Ado, adenosine; ADR, adenosine receptor; ARPP-16, cAMP-regulated phospho-protein 16; CaM, calmodulin; D1R, dopamine receptor type 1; D2R, dopamine receptor type 2; DA, dopamine; DAT, dopamine transporter; DARPP-32, dopamine and cAMP-regulated phospho-protein 32; DDC, dopa decarboxylase; Dopa, 3,4-dihydroxyphenylalanine; DOPAC, 3,4-dihydroxyphenylacetic acid; Glu, glutamate; HCN, hyperpolarization-activated cyclic nucleotide-gated ion channels; HVA, homovanillic acid; NMDA, N-methyl-D-aspartate receptor; NO, nitric oxide; NOS, nitric oxide synthase; MAO, monoamine oxidase; mGlu, metabotropic glutamate receptor; PDEx, phosphodiesterase type x; PPx, protein phosphatase type x; PKG, cGMP-dependent protein kinase; sGC, soluble guanylate cyclase; TH, tyrosine hydroxylase; Tyr, tyrosine; VMAT, vesicular monoamine transporter. Black arrows mean direct synthesis or transport, black lines with circled end mean direct positive (+) or negative (-) influence or degradation, dotted arrows mean indirect positive (+) or negative (-) influence. Panel **(A)** was created in BioRender.com and the rest of the figure with Microsoft PowerPoint.

Methylphenidate and amphetamines are stimulant drugs that are first-line pharmacotherapies for ADHD (13). These drugs block DA and norepinephrine (NE) transporters, inhibiting catecholamine reuptake into dopaminergic nerve terminals (**Figure 1**), increasing their availability at dopamine receptors. Stimulants can also interact with other target molecules and transmitter systems, e.g. amphetamines can increase DA release from synaptic vesicles, and inhibit monoamine metabolism, particularly at high doses (14). Stimulants have effects on ADHD symptoms, possibly by optimizing DA and NE modulated task-related brain networks that increase perceived saliency, reducing interference from the default mode network (15).

Although specific NE transporter inhibitors and alpha-2a adrenergic agonists are also effective against ADHD symptoms (16), recent meta-analyses show that psychostimulants also acting on dopaminergic pathways, like methylphenidate and amphetamines, have greater effect sizes for ADHD symptom management compared to NE transporter inhibitors like atomoxetine or the alpha-2a agonist guanfacine (13). Some arguments against the mono-causal dopamine-deficit hypothesis such as that by Gonon (17) have cited evidence that psychostimulants improve behavior, including attention, in healthy children as well as those with ADHD (18, 19), the rodent models agreed to have the highest validity for ADHD (20) still cannot closely fit the neuropharmacology of ADHD (see (17)), and meta-analyses of brain imaging studies do not support the idea that ADHD symptoms can be explained by hypo-function within a few isolated brain regions (21). Some more recent skepticism of the hypothesis comes from highlighting the shortcomings of the dopamine-focused executive dysfunction theory in explaining hyperactivity and motor problems in ADHD (22). Conversely, recent meta-analyses continue to show that other dopamine-related cognitive dysfunctions, such as impaired inhibitory control, are robustly exhibited in ADHD [e.g. (23)].

Different versions of the DA hypothesis of ADHD have been around for decades and reviews dating as far back as 1991 (24) have collated studies arguing for and against the key role of DA in this condition. Since then, the link between DA and ADHD has been further investigated using a variety of approaches. By way of illustration, a literature search using the PubMed database in August 2024 retrieved 184,551 hits with the key word “dopamine”, 51,665 with “ADHD”, 2,990 with the search terms “dopamine”, “attention deficit hyperactivity disorder” and “ADHD”, including 39 meta-analyses. The main focus of these meta-analyses is on genetic studies, often exploring one or a few genetic markers in a single candidate gene. Across this extensive literature there is no clear consensus what the DA hypothesis of ADHD specifically refers to – either that DA plays a key role, there is a hypo-functioning DA system, there is a hypo-dopaminergic state in specific brain regions like the prefrontal cortex (PFC), there is a hypo-dopaminergic state throughout frontostriatal regions, or specifically that extracellular DA concentration/DA signaling/DA metabolism is lowered. *For the purposes of this review, we refer to the DA hypothesis as: reduced synthesis, content, metabolism and rate of extracellular accumulation of the DA neurotransmitter within frontostriatal regions in people with ADHD*.

Considering the large number of studies published on the connection between DA and ADHD, spanning >40 years of research using a diversity of methods - including different types of brain imaging, pharmacology, neurochemistry and genetic studies in ADHD patients, healthy humans and animal models - it is practically impossible to systematically explore and extensively review all the evidence collected during this period. The purpose of this review is rather to examine how far we have come in the past >40 years by evaluating different lines of evidence for the DA hypothesis. We take a multidisciplinary approach and extend our focus beyond genetics to also examine the DA hypothesis from a neurophysiological, neuropsychological, and neurometabolic perspective, attempting to cite representative and/or recent developments in these areas using publications entered into the PubMed database before September 2024 as the primary source of information. Major reviews and controversial reports have been discussed among the author group before being included. In this way we aim to provide an updated overview of the main aspects of the DA hypothesis in ADHD. Although we have strived to present a balanced and representative overview, we did not aim to include every possible perspective or all published reports in these fields. Inevitably, some aspects and data will unintentionally be overlooked or underrepresented in such a large and rapidly evolving research field.

We start by providing a brief overview of the dopaminergic system covering DA synthesis, receptors and downstream signaling mechanisms, while highlighting some points of complexity that may help to explain why the DA hypothesis is still so hotly debated.

## An overview of the dopaminergic system

DA is a neurotransmitter/neuromodulator that plays crucial roles in several physiological processes in the brain and peripheral tissues. The brain functions include motor control, cognition, learning, food intake and reward (25, 26). Peripheral DA is implicated in the immune system, hormone release, gastrointestinal motility, regulation of blood pressure and sodium balance and probably other functions (27). The main components of the dopaminergic system include the biosynthetic machinery involved in DA synthesis and release, pre- and postsynaptic DA receptors, as well as downstream signaling mechanisms and enzymes and transporters involved in DA metabolism (25) (**Figure 1**). DA is synthesized from the amino acid tyrosine through two enzymatic reactions involving hydroxylation and decarboxylation of this precursor amino acid into a monoamine. Tyrosine hydroxylase (TH) catalyzes the first rate-limiting step in this process (28, 29) and dopa decarboxylase (aromatic amino acid decarboxylase, DDC) catalyzes the second step. Once synthesized, DA is stored in vesicles within the dopaminergic neurons. Upon neuronal activation, DA is released into the synaptic cleft or extrasynaptically (volume transmission) where it interacts with receptors on target cells.

### Dopamine homeostasis

Although neuronal communication is highly dynamic on a temporal scale, the concept of neurotransmitter homeostasis is still used. For rapidly acting transmitters this refers more to the signaling capability or the transmission process rather than the synaptic transmitter levels themselves. For more slowly acting neurotransmitters, like the neuromodulator DA, it makes more sense to also talk about the extracellular transmitter homeostasis – particularly for the volume transmission. Thus, neurotransmitter homeostasis is a result of transport processes between different compartments such as release, diffusion and reuptake, but also relies on the biosynthetic and metabolic fluxes acting locally in the region of interest. Presynaptic regulation is key to understanding these processes. However, the postsynaptic signaling responses are of course important for the neurotransmission homeostasis as a whole. Both signaling systems will be briefly discussed here.

Dopaminergic neurons of the central nervous system (CNS) have anatomically highly complex axons with extensive arborizations in their target areas of the brain – extending from their nuclear localization (substantia nigra pars compacta and ventral tegmental area) to large areas of the brain (30). There they form two general types of contacts; varicosities, which are enlargements of the axon that contain secretory vesicles and mediate diffuse modulatory stimuli on the surrounding neurons, and more defined synaptic contacts. For volume secretion from diffuse varicosities, reuptake is expected to be of little importance for temporal changes in the cytoplasmic dopamine levels as extracellular diffusion of dopamine is expected to be rapid (30). The frequency of release does not directly reflect the depolarization dynamics of the axon. These neurons are known to operate in different firing modes: low frequency tonic mode (~5 Hz) and transient phasic high frequency or bursting (< 30 Hz). The amount of DA released is much higher during phasic firing and is further modulated by local signaling inputs such as glutamine, gamma-aminobutyric acid (GABA), and acetylcholine. DA release and its temporal concentration profile has been extensively studied and measured quantitatively in many brain regions. Terminal release is modulated by DA-autoreceptors, which modulate the amount of DA released during depolarization. Somatodendritic release of DA modulates the activity of the neuron itself (31).

There are considerable differences between different brain regions in their turnover and reuptake of DA. High levels of the dopamine transporter (DAT) are found in the terminal regions of the nigrostriatal and mesolimbic pathways, whereas low levels are found in somatodendritic regions and the PFC (32). The different reuptake activities have a considerable impact on the volume to which DA spreads and the temporal profile of the transmission.

### Dopamine receptors

DA exerts its effects by binding to specific G protein-coupled receptors (GPCRs) (**Figure 1**). There are five known human DA receptor subtypes: D1, D2, D3, D4, and D5 (33, 34). D1-like receptors (D1 and D5) activate adenylyl cyclase, leading to increased cyclic AMP (cAMP) levels. D2-like receptors (D2, D3, and D4) inhibit adenylyl cyclase and modulate cAMP levels (34). These receptors are widely expressed in both the CNS and peripheral tissues. In addition to regulating levels of cAMP, DA receptors can activate other signaling pathways, including the Gq/11 mediated activation of phospholipase C (PLC) (35), β-arrestin 2 induced activation of Akt, and transactivation of tyrosine receptor kinases such as TrkB (36).

Dopamine receptors (DRs) are found presynaptically on DA neurons and postsynaptically on different target cells (33) (**Figure 1**). DA signaling complexity is further increased by formation of oligomeric complexes between different types of DRs, as well as complexes with other GPCRs and other receptor molecules. Reported DR heteromers include D1/D2, D1/D3, D2/D4, D2/D3 and D2/D5 DR heterodimers, as well as D1/D2/D3 + adenosine A1 or A2 receptors, D2 + somatostatin SST5 receptors, D2 + 5HT2A receptors, D1 + metabotropic glutamate receptors (Glu5), D1 + Histamine H3 receptors, D2 + Histamine H3 receptors, D2 or D3 + glutamate NMDA receptors (NR2B) (37–39). Some of the heteromeric receptor complexes have been characterized, showing that their DA binding and effector coupling profiles are distinct from the respective homodimers or monomers (39). Still, the evidence for the *in vivo* formation and physiological relevance of most of the reported oligomeric complexes between DRs is limited and even considered controversial (38, 40).

Most D1 and D2 receptors are expressed on non-dopaminergic neurons and are involved in a diversity of brain functions, including learning, cognition, motivation and motor functions. D1-like receptors exert these functions partially by modulating ion channels, such as voltage gated sodium, potassium and calcium channels, as well as G-protein coupled inwardly rectifying potassium channels (GIRKs) (41, 42). D2-like receptors are coupled to Gi/o proteins and thereby inhibit GIRKs. Presynaptic D2 autoreceptors modulate DA functions *via* activation of potassium conductance, as well as by downregulating the activity of the plasma DA transporter and the activity of TH (43, 44) (**Figure 1**). As D2 receptors are the primary targets of antipsychotic drugs, the physiology and pharmacology of D2 subtype receptors have been extensively studied. However, due to the strong structural and functional interconnectivity of the dopaminergic system and other neurotransmitter systems, in particular other monoamines, it is virtually impossible to completely disentangle the “true” dopaminergic neurotransmission from other transmitter systems (37).

### Dopamine during brain development

DA production starts early during embryonic brain development, even before synapses are formed and DA exerts its functions at critical periods during development (45). For example, D2 receptors are involved in long term depression (LTD) and synaptic pruning during development of the rat anterior cingulate cortex (46). The hyperactive anterior cingulate cortex observed in *Drd2*+/- rats is believed to drive anxiety-like behaviors and perhaps also other behavioral symptoms (46, 47). Although different DRs are expressed during embryonic development, their patterns of expression are shifting during fetal and postnatal development, reaching a stable level at adulthood (45). This delayed maturation of DRs during development implies that the DA system also is subject to multiple genetic and environmental factors. Altered DA functions during neurogenesis have been linked to several neuropsychiatric/neurodevelopmental disorders including autism, schizophrenia and ADHD (45).

### Dopamine transporters

DAT is responsible for reuptake of DA from the extracellular space back into presynaptic neurons or other cell types. Together with the related monoamine transmitter transporters, such as the norepinephrine transporter (NET) and serotonin transporter (SERT), human DAT belongs to the family of neurotransmitter sodium symporter family of transporters, which are part of a large family of solute carrier 6 (SLC6) transporters (**Figure 1**). The human DAT, NET and SERT proteins are encoded by *SLC6A3*, *SLC6A2* and *SLC6A4*, respectively (48). Inhibition of DAT by stimulant drugs like cocaine and amphetamines increases extracellular DA levels. Many natural monoamines and synthetic drugs bind with variable affinity to all these transporters, which can be predicted by their strong amino acid sequence conservation (49).

While DAT is responsible for transporting DA across the plasma membrane, another class of transporters, the vesicular monoamine transporters (VMATs) are responsible for the accumulation of DA and other monoamines (e.g. serotonin, NE and histamine) in synaptic vesicles (50) (**Figure 1**). VMAT2 (encoded by *SLC18A2* in humans) is the major vesicular monoamine transporter for DA in the adult brain, while VMAT1 is mainly expressed during embryonic development and in peripheral tissues. Mice lacking VMAT2 die soon after birth, while heterozygotes develop normally, but have increased sensitivity to amphetamine (51).

### Dopamine metabolism

The enzymes monoamine oxidase (MAO) A and B and catechol-O-methyltransferase (COMT) metabolize DA and similar monoamine substrates. MAOs are found in the outer membrane of mitochondria in most cell types (**Figure 1**). In contrast, COMT acts both intracellularly and extracellularly, as the membrane bound form of this enzyme is oriented with its C-terminal catalytic domain facing the extracellular space (52).

### Presynaptic regulation

TH is the key regulatory enzyme in DA biosynthesis and its activity is under regulation by several mechanisms (28). Thus, availability of its substrates (tyrosine (Tyr), tetrahydrobiopterin (BH4) and oxygen), feedback inhibition by catecholamines, active-site iron redox-state, serine (Ser)-phosphorylation, protein-protein interactions, protein localization, turnover and expression all regulate TH activity in the different neuronal compartments (53, 54). Tyr availability is governed by transport across the blood brain barrier and is influenced by the level of other amino acids that compete for the same transport mechanism (55). TH is also characterized by extensive substrate inhibition of Tyr, which could compromise its activity in conditions with significantly elevated Tyr levels (56). The activity of TH is shut down *via* feedback inhibition from high affinity binding of catecholamines to the active site iron, but the enzyme can be reactivated by phosphorylation at Ser40 (57). The cAMP dependent protein kinase (PKA) is a prominent TH-Ser40 kinase in the brain that is further regulated by D2 autoreceptors (57). There are several signaling pathways that regulate TH phosphorylation on Ser19, 31 and 40, locally in the striatum and likely in other brain regions (57–59). Ser19 phosphorylation enables binding of TH to the 14-3-3 proteins, which regulate activity, secondary protein interactions and multi-site phosphorylation of the enzyme (57, 60). Much less is known about the regulation of other proteins in the DA synthesis pathway. However, there are reports of phosphorylation-mediated regulation of DDC, VMAT2 and DAT (61, 62).

### Regulation of dopamine signaling

The cyclic nucleotide signaling pathways are central to DA signaling. The activation or inhibition of cAMP signaling is affected by receptor configurations and the expression and protein regulation of downstream components. The adenylate cyclases and guanylate cyclases that synthesize cyclic nucleotides and the phosphodiesterases (PDEs) that degrade the cyclic nucleotide second messengers, are important in shaping downstream DA signaling. PDEs in particular are well explored drug targets (63, 64). The mammalian PDEs comprise a superfamily of 11 members, originating from 21 different genes that give rise to > 100 different isoforms (65). These are differentially expressed in different tissues and have diverse substrate selectivity (towards cAMP and cGMP), regulation, and cellular localization. Brain regional differences in PDE isoform expression have been reported for several of the PDE genes. For example, the dual specificity PDEs PDE1B1 and PDE10A are highly expressed in striatal medium spiny neurons (MSN) (63, 64). Knock out mice of the Ca2+/calmodulin stimulated PDE1B show increased locomotor activity and amphetamine sensitivity. The opposite has been reported for inhibition of PDE10A (66). The PDE10A has high affinity for cAMP and has been shown to be important for maintaining baseline cAMP levels and PKA activity in striatal MSN (67). The cGMP activated PDE2A has been found to be mainly located in the axonal and terminal compartments of pyramidal neurons in the cortex and hippocampus, striatal MSN, and in the medial habenula. The terminal location was confirmed by high immunoreactivity in the globus pallidus, substantia nigra pars reticulata, and interpeduncular nucleus (68). The presence of this PDE in the terminal compartment would contribute to nitric oxide mediated inhibition of presynaptic activity as cGMP will stimulate PDE2A and decrease the cAMP levels. For the dopaminergic terminals, less is known about the key PDEs that control PKA activation and thus TH Ser40 phosphorylation. So far, only members of the PDE4 family have been put forward, based on inhibitor studies (69). More research is needed to understand the key players in DA signaling at the cellular detail of different brain regions.

Together these examples illustrate some of the extensive signaling diversity that can be generated even in a simple signaling pathway such as cAMP signaling. There are many additional layers of complexity as illustrated in **Figure 1**. The involvement of these signaling modules across multiple pathways and biological functions also implies that it is virtually impossible to define a single “DA-specific” biological system.

## Genetic perspective

### Animal models

Widely studied animal models of ADHD include spontaneously hypertensive rats (SHR), DAT knockout (KO) mice and Coloboma mice. The latter model has a mutation in the SNAP-25 gene, affecting synaptic vesicle release and resulting in hyperactivity and attention deficits (70). As SNAP-25 has multiple functions and affects many transmitters and ion channels, the superficial phenotypic resemblance of Coloboma mice to ADHD can hardly be attributed only to the DA system. SHRs exhibit many metabolic alterations, hypertension, hyperactivity, impulsivity, attention deficits, and altered monoamine levels, but the exact origin of this complex phenotype is still being debated. Several of the known proteins and genes involved in DA signaling have been studied in rodents, zebrafish and fruit fly models, which have partially confirmed their role in ADHD-like behaviors. A recent review listed 111 different genetically modified mouse strains implicating 103 different causal genes that show “exclusively ADHD-related behaviors” (70). Many of these genes have also been implicated in trans-synaptic signaling and forebrain development, including but not limited to dopaminergic synapses. Genetic variants in transcription factors (e.g. FOXP1, FOXP2 and MEFC2) are among the most robustly associated genetic findings in ADHD, autism and other neurodevelopmental disorders (see below) (71, 72). The forkhead box (fox) transcription factors FOXP1/FOXP2 regulate expression of many downstream proteins involved in neurogenesis, synaptic development and functions and are highly expressed in neurons involved in striatal dopaminergic signaling (73). Loss of *Foxp1* and *Foxp2* results in impaired motor and social behavior in mice, possibly by altering potassium channel functions (**Figure 1**) (73). This example illustrates the complexity of the dopamine system, how different animal models can be used to explore various aspects of these functions and how they relate to human genetic findings. However, it has also been shown that different animal models with different genetic backgrounds can give conflicting results (70), which demonstrate the complexity of animal (and human) behavior and the inherent limitations of animal models. For a summary of animal studies exploring monoamine functions in ADHD models, see Viggiano et al. (74). Although many genetic ADHD models with altered DA signaling have been examined, there is probably not a single animal model that can adequately recapitulate all clinically relevant aspects of this complex human phenotype (70).

Animal models, particularly DAT, NET and SERT KO mice, have also been used to elucidate the molecular targets of ADHD medications. Mice lacking DAT were reported to show hyperlocomotion and indifference to cocaine and amphetamine (75), but self-administration of cocaine persisted in these DAT-KO mice, possibly indicating involvement of multiple targets (e.g. NET and SERT) and compensatory mechanism in such animal models (76). A recent study compared the behavioral effects of monoamine transporter drugs on DAT-KO and NET-KO mice (77). The NET blocker desipramine increased PFC DA and NE levels in DAT-KO but not in NET-KO mice. Furthermore, desipramine or the dopamine beta-hydroxylase (DBH) inhibitor nepicastat reduced hyperactivity in DAT-KO mice. DBH catalyzes the conversion of DA to NE so inhibition of DBH depletes the brain of NE, while increasing levels of DA. Based on these observations, the researchers concluded that increased PFC levels of DA rather than NE was the common mechanism explaining the ‘paradoxical’ calming effects of psychostimulants. However, these animal models also show developmental compensatory effects, such as alterations in striatal D2 receptors in DAT KO mice, that could contribute to the phenotype (78).

### Human studies

Although twin studies have shown that ADHD in children and adults has a high heritability (79), it is considered a polygenic disorder, and it has been difficult to find consistent genetic markers for this trait (80). Since the 1980s linkage, candidate gene, genome wide association (GWA), whole exome sequencing and whole genome sequencing studies have been used to study the molecular genetics of ADHD (80–82). As stimulants target DATs and other monoamine transporters, like SERT, NET and VMAT2, the genes encoding these transporters, as well as the five human DRs (*DRD1-5*), have been intensively explored in candidate gene studies (83–87). Genetic variants of *DRD4* have been investigated particularly intensively in connection with ADHD (88, 89). In 1993 it was reported that the *DRD4* gene has a hypervariable segment (VNTR) encoding an intracellular domain of the DA receptor (90). The frequency of the different 4R, 7R, and 2R alleles varies widely across ethnic groups. For instance, the prevalence of the 7R allele was reported to be <2% in Asian populations and ∼48% in native Americans. It was suggested that these differences could be due to positive selection (91), but later studies have not confirmed a positive selection favoring the 7-repeat allele of VNTR in the *DRD4* gene. Instead, the increased population frequencies of the *DRD4* 7R allele in African and European populations can be explained by random genetic drift (92, 93). Although recent meta-analyses and original studies in various populations show possible associations with *DRD4* variants in clinical subgroups of ADHD, no unifying or generally accepted hypothesis for the role of *DRD4* in ADHD has been presented (94, 95).

Early candidate gene studies also suggested associations between various genetic markers in the other *DRDs*, *DAT1* (*SLC6A3), COMT*, and *DBH*, but later meta-analyses have given mixed results (88, 89, 96–98). In a comprehensive meta-analysis, Gizer et al. reported significant associations for *DAT1, DRD4, DRD5, 5HTT, HTR1B*, and *SNAP25*, but also significant heterogeneity for the associations between ADHD and *DAT1, DRD4, DRD5, DBH, ADRA2A, 5HTT, TPH2, MAOA*, and *SNAP25* (88). Many subsequent studies have since investigated these candidate genes in different populations, reporting nominal associations in clinical subgroups with different genetic markers in some of the genes (94, 99). Compared to recent GWA studies (see below), these studies were all based on small clinical samples (hundreds of individuals) with variable reporting practices and borderline significant results, compared to tens of thousands of individuals, harmonized genotyping platforms and protocols and stringent significance levels used in recent GWA studies. In the absence of sufficiently powered and replicated studies, e.g., on the *DAT1* or *DRD4* VNTRs, there is still some uncertainty regarding the robustness of these findings and the role of individual genetic variants in core DA related genes in ADHD.

Unlike candidate gene studies that have a limited focus, GWA studies aim to investigate common genetic markers across the entire genome. Due to the polygenic nature of ADHD, early GWA studies did not provide any genome wide significant findings. However, using a sample of >20 000 ADHD cases and >35 000 controls, the first 12 genome wide significant hits were published in 2019 (71). When this sample was expanded to >38 691 cases and 187 843 controls, 27 significantly associated signals were obtained, many of which could be linked to credible functional variants and risk genes (72). None of the previously nominated candidate genes, including DA related genes or known drug targets for ADHD, appeared among the top ranked risk genes (100). Among the core DA related genes, *DRD2* appeared promising, but not at the genome wide significance level. Human *DRD2* is found in a gene rich region on chromosome 11. Some of the most studied *DRD2* variants are found in neighboring genes, such as the common variant rs1800497, which is located downstream of *DRD2* within the ankyrin repeat of *ANKK1* that encodes a Ser/Thr protein kinase. The rs1800497 variant correlates with expression levels of *DRD2* and has been associated with multiple psychiatric and somatic disorders and common traits. The rs1800497 and many other common variants in proximity to the *DRD2* locus are significantly associated (p< 10^-8^ in GWA studies) with smoking (101), alcohol consumption (102), risk tolerance (103), schizophrenia (104), depression (105), suicide attempts (106), neuroticism (107), educational attainment (108), as well as cross-disorder (103) psychiatric disorders (8 diagnoses) (109). However, it is not clear whether these phenotypes are mediated by the *DRD2* protein or by other neighboring genes or regulatory DNA sequences. In comparison to these well-established associations, the connection of psychiatric disorders and behavioral traits with other DR genes are much less consistent, although there are some GWA studies showing that *DRD1* is associated with educational attainment (108) and *DRD3* with neuroticism (110). As the DRD2 receptor protein is expressed at higher levels than other DRs in the human brain (Human Protein Atlas) and has important autoregulatory functions (**Figure 1**), it is not surprising that *DRD2* variants also show the strongest associations with human disease and behavioral traits. However, in comparison to the cited associations with other traits, the largest GWA study of ADHD did not show genome significant associations with *DRD2* variants or any other core DA related genes (72).

The failure to replicate candidate gene studies, e.g. the *DAT1* or *DRD4* VNTRs, in large GWA studies of ADHD could be due to the poor tagging of such variants (i.e. not being in linkage disequilibrium) with SNPs typically included in GWAS. Other explanations could be that the patient and control samples included in the early reports were different from more recent and larger GWA studies or due to publication bias where some early positive studies have dominated the literature. Furthermore, the lack of genome wide significant associations (p <10^-8^) with core DA-related genes could still be a statistical power issue, as the ADHD sample sizes are still relatively small compared to those used for some other complex traits. Although modest associations were found for the core DA genes, pathway analyses showed enrichment in dopaminergic gene transcripts (72). Overall, it seems clear that genetic alterations in DA-related genes are found across several behavioral traits but that ADHD does not currently stand out as one disease uniquely or predominantly associated with such genetic variants.

Early candidate studies in ADHD and other traits often prioritized coding variants with functional effects. In contrast, GWA studies typically explore common, noncoding variants with unclear functional effects of their own. A study comparing rare and common variants in early and late diagnosed ADHD found that childhood ADHD had stronger genetic overlap with hyperactivity and autism compared with late-diagnosed ADHD and the highest burden of rare protein-truncating variants in evolutionarily constrained genes (111). As more whole exome sequencing and whole genome sequencing data become available, we expect that a clearer picture of phenotypic correlates of different human genes and variants will emerge across the full spectrum of common and rare genetic variants.

In addition to analyzing single genetic variants, it is possible to collapse multiple variants into a single gene or analyze many related genetic variants and genes together, e.g. multiple genes in the dopaminergic pathway, using MAGMA or similar software tools (112, 113). In a review from 2003 it was concluded that “*SLC6A3* and *DRD4* genes in ADHD appears to be one of the most replicated in psychiatric genetics and strongly suggests the involvement of the brain DA system in the pathogenesis of ADHD” (114). However, as detailed above, not all subsequent data have supported this conclusion. Such early gene set analyses were limited by small sample sizes and the low number of genetic variants available for testing. With access to GWA data it has been possible to perform gene set and pathway analyses more systematically and at a larger scale.

In 2022 Cabana-Domínguez et al. published a gene set analysis using publicly available GWAS data and manual curation to explore combined genetic signals from DA and serotonin (DA core with 12 genes, and SERT core with 23 genes) (115). The analysis contained the well-known dopaminergic or serotonergic genes (neurotransmitter receptors: *DRD1-5*, 5-hydroxytryptamine receptor (*HTR)1A-B*, *HTR1D- F, HTR2A-C, HTR3A-E*, *HTR4*, *HTR5A*, *HTR6*, *HTR7*; transporters: *DAT1/SLC6A3*, *5HTT/SLC6A4 (*serotonin transporter gene*)*; and related enzymes *DDC*, *TH*, tryptophan hydroxylase (*TPH)1*, *TPH2*, *DBH*, *COMT*, *MAOA*, *MAOB*). Using the MAGMA software for gene-based analyses, no significant multiple testing corrected association was observed with either the DA or SERT gene sets and ADHD, but the SERT genes were weakly nominally associated with ADHD. However, when an expanded list of 265 “wider dopaminergic genes” using Gene Ontology and KEGG pathway databases (as updated in 2016) was tested, they found significant associations for ADHD and autism spectrum disorder (115). Notably, a major caveat of such studies is that few of the included genes or genetic variants are uniquely associated with dopaminergic neurotransmission. For example, the genes and proteins involved in DA synthesis, storage, release, and degradation are also involved in synthesis of other neurotransmitters such as NE, serotonin, and nitric oxide. As detailed above, even the DA receptors are known to interact with multiple transmitters. Cabana-Domínguez et al. noted that 20% of the genes in the DA set were also present in the SERT set and 32% the other way round, but this estimate may be too low (115).

### Gene-environment interactions

Low birth weight, preterm birth and perinatal asphyxia are among the best documented environmental risk factors of ADHD (116, 117). Fetal hypoxia can result in neuronal death, white matter damage and reduce the growth of neural processes (118). Perinatal hypoxia and exposure to neurotoxins such as 6-hydroxydopamine and MPTP (1-methyl-4-phenyl-1,2,3,5-tetrahydropyridine) have also been shown to reduce brain DA syntheses in animal models (119, 120). As human TH is also very sensitive to reduced oxygen tension, it is likely that DA synthesis is also reduced in the human brain under hypoxic conditions (54).

The generation and maintenance of dopaminergic neurons also requires a precise transcriptional cascade at specific time points during brain development. For instance, *Fox1a*, *Foxa2* and *Nurr* promote a dopaminergic phenotype in the mouse brain (121). However, most of these transcription factors are also expressed in other cell types and transmitter systems. The DA neuron specifying transcription factors could be promising risk genes for psychiatric disorders that should be more systematically explored. As many risk genes for neuropsychiatric disorders are mainly expressed during embryonic development, it is possible that any putative altered dopaminergic functions in ADHD patients could be present before birth, but not necessarily in children or adults. Furthermore, it might be possible that the causative genes are not among core dopaminergic genes, but rather in other developmental or regulatory factors.

As mentioned above, alterations in the DA system can be secondary to other genetic or environmental changes in transcription factors or other proteins involved in neurodevelopment. Linkage studies in large multigenerational families in Colombia led to the discovery of the latrophilin 3 gene *LPHN3* (*ADGRL3*) as a risk gene in ADHD (122, 123). Latrophilins 1-3 are postsynaptic adhesion G protein-coupled receptors that have been implicated in brain development and synaptic maturation (124) and linked to developmental behavior abnormalities in rodents and humans (125). Animal studies have shown that Latrophilin 1 or 3 haploinsuffiency (*ADGRL1* or *ADGRL3* knockouts) lead to developmental abnormalities and altered dopamine signaling (126). Similarly, the Wnt/mTOR (wingless-related integration site/mammalian target of rapamycin) pathway has been implicated in ADHD based on genetic and pharmacological evidence (127). The Wnt-signaling pathway is involved in cellular proliferation and differentiation and mTOR has been implicated in neurodevelopment, dopamine signaling and synaptic plasticity (128). Interestingly, exposure to manganese in neonatal rats results in attention deficits, dysregulation of mTOR signaling and downregulation of DA synthesis in brain prefrontal cortex (129). These examples show that alterations in brain DA functions may be due to a primary genetic predisposition, environmental exposures such as pharmacological agents, or specific interactions between genes and the environment (130).

Concluding remarks: Many different animal models, including some models with altered DA function, show phenotypic resemblance to ADHD, including hyperactivity and inattention. However, it is unclear whether these behaviors are specific and how they can be used to trace the biological origins of human ADHD. Although the DA-system is indirectly implicated in several of the most robustly associated ADHD risk genes, associations with the core set of genes involved in DA synthesis, DA receptors or transporters are less consistent. There is also a need for larger and more diverse human samples, as well as for integrative approaches that combine genetic and environmental data in order to provide a more comprehensive perspective on ADHD neurobiology.

## Neurometabolic perspective

Recent genetic advances have revealed risk genes for hundreds of different diseases and traits, including genetic causes of recessive lethality (131). Nearly 5000 human genes are known to exist as homozygous complete knockouts in the human population (131). Phenotypic characterization of such genetic variation constitutes a rich source of information that is highly relevant for studying normal gene function, rare genetic syndromes, as well as common, polygenic and complex human disorders, such as ADHD. Thus, rare monogenic syndromes may reveal novel biological functions of the affected gene(s) and also shed light on the role of these genes in common complex disorders (132). Disrupted brain metabolism as a potential causative element to the ADHD phenotype is worth exploring from this perspective.

Inborn errors of metabolism constitute a subgroup of “genetic” diseases that typically affect enzymes, transmembrane signaling proteins or co-factor synthesis relevant to the function of key enzymes (133). The International Classification of Inherited Metabolic Disorders (134) currently recognizes over 1,450 conditions, hundreds of which belong to the heterogeneous and expanding group of inherited neurometabolic disorders (NMDs;//www.omim.org/) that affect metabolic processes relevant to brain function. The immediate molecular consequence of such enzyme deficiencies – whether they are present within the CNS or occur peripherally – may present as a decrease/absence of critical metabolites and/or the accumulation of toxic intermediates. Such metabolic defects typically interfere with cellular processes involved in neuronal signaling or produce tissue damage, potentially affecting multiple organs. Human Mendelian “model” diseases have been characterized for virtually every known gene and protein involved in monoamine functions. Some of these genes and conditions are briefly reviewed here. As for animal models, interpretation of the phenotypic consequences of genetic variants, including complete loss of function (LOF) variants, must consider early (prenatal) developmental effects, as well as redundant, overlapping and compensatory mechanisms of different genes.

Human variants of *DAT1 SLC6A3* are of particular interest in the context of ADHD. Infants with early onset *SLC6A3*-related DA transporter deficiency syndrome typically manifest nonspecific findings with irritability, feeding difficulties and hypotonia and delayed motor development, but not typical ADHD symptoms. In contrast, these children typically show a “neurological” type of hyperkinetic movement disorder with chorea, dystonia, ballismus and orolingual dyskinesia and later also show typical neurological symptoms (135).

Similar observations have been made for children and adults with severe defects in DA synthesis. LOF mutations in *DDC* - the gene encoding the enzyme immediately responsible for DA synthesis - have been associated with severe neurodevelopmental delay, hypotonia, oculogyric crises, and a complex movement disorder with autonomic features, but again not typical ADHD symptoms (132, 136). Since DDC has a broad range of substrates and is involved in the synthesis of other monoamines (histamine, serotonin, NE), such symptoms are not necessarily related to altered dopaminergic functions and could also be due to early prenatal neurodevelopmental alterations. As the enzyme TH is a specific marker of catecholaminergic neurons and the rate limiting enzyme in DA synthesis, it has also been explored in the context of ADHD. Central TH deficiency, resulting in mild-to-severe DA deficiency, has been associated primarily with movement symptoms - or in severe cases, progressive encephalopathy with mental retardation – but apparently no reports of ADHD symptoms (5). For comparison, as Parkinson’s disease is characterized by very low levels and dysregulated TH, it has been termed a TH deficiency syndrome (28). LOF mutations of *DBH* typically present with very low levels of NE and severe orthostatic hypotension and eyelid ptosis, but normal intellectual development and no record of ADHD symptoms (137). Animal studies show that total lack of DA or NE is incompatible with life, but less severe missense variants in DBH may be beneficial as they are protective against age related arterial hypertension (138).

Phenylalanine, tyrosine, and tryptophan are aromatic amino acid (AAA) precursors to the production of monoamine neurotransmitters such as DA, NE, adrenaline, serotonin, and melatonin. Deficiencies in the enzymes governing the conversion and breakdown of AAA precursors result in various metabolic disorders categorized as aminoacidopathies. Associations have been observed between aminoacidopathies, which purportedly result in low prefrontal DA levels, and neuropsychiatric disorders, including ADHD (139). Specifically, tyrosinemia type 1 (TYRSN1) and phenylketonuria (PKU) have been linked to core symptoms of ADHD, mainly presenting as impaired executive functioning in affected patients (140, 141). Shared pathophysiological mechanisms have been suggested (139, 140), partly supported by the improvement of cognitive symptoms in response to amphetamine treatment (142). However, in treated TYRSN1 there is debate over whether elevated tyrosine levels lead to increased or attenuated DA synthesis (140, 143). Furthermore, not all patients respond positively to stimulants, and the general response in healthy adults to these drugs is rather similar (see above).

The synthesis of DA and other transmitter molecules also depends on an adequate supply of enzyme cofactors, i.e. tetrahydrobiopterin (BH4) for TH, pyridoxal phosphate (vitamin B6) for DDC and ascorbate (vitamin C) for DBH (132). A lack of tetrahydrobiopterin is the most common and best-established genetic cause of DA deficiency in humans. LOF mutations in genes encoding the BH4 synthetic enzymes are found in the autosomal recessive neurological disorder DOPA responsive dystonia (Segawa syndrome) (144). This condition is clinically similar to TH deficiency and characterized by low levels of homovanillic acid and 5-hydroxyindoleacetic acid in CSF, microcephaly, early onset neurological symptoms, but not typical ADHD or other psychiatric symptoms or disorders (145). Although BH4 is also an essential cofactor for synthesis of serotonin, nitric oxide and other metabolites, the clinical picture is dominated by hyperphenylalaninemia and DA deficiency (145). As the cofactor for DDC (pyridoxal phosphate, a derivative of pyridoxamine, vitamin B6) is needed for hundreds of different enzymes and metabolic pathways (146), vitamin B6 deficiency has multiple manifestations from different organ systems and has been associated with increased risk of diabetes, heart disease, and cancer (147). Although again, ADHD is not among the defining symptoms in these conditions of DA deficiency. Similarly, severe ascorbate deficiency interferes with NE synthesis *via* DBH and causes scurvy, which is a syndrome primarily affecting connective tissue but apparently with less consequences for brain function (148).

Concluding remarks: Hundreds of patients with different monogenic diseases that lead to abnormally low DA levels in the brain have been characterized. The clinical features reported for these patients are dominated by motor neurological symptoms, not by ADHD or other psychiatric disorders. Although these observations do not directly implicate low DA levels as the defining feature in ADHD, partially overlapping cognitive deficiencies with aminoacidopathies and related conditions suggest that aspects of disrupted monoaminergic brain metabolism could contribute to the broader clinical picture of ADHD.

## Neurophysiological perspective

Positron emission tomography (PET) and single photon emission computerized tomography (SPECT) can measure neuronal activity indirectly through measurements of metabolism, blood flow, and ligand–receptor interactions. Studies using these methods are therefore worthwhile examining in more detail as they can visualize neurotransmission *in vivo* and provide relatively strong support for/against the DA hypothesis.

One of the earliest ADHD PET studies suggesting decreased extracellular DA levels was a 1999 preliminary study with children by Ernst and colleagues (149). Extracellular DA was decreased in the midbrain but not the striatum or frontal regions. Despite the high citation count, the authors acknowledged that the main finding of decreased extracellular DA in the right substantia nigra/ventral tegmental area compared to healthy controls did not survive Bonferroni correction. In theory, a higher [^18^F]DOPA uptake reflects elevated presynaptic AADC activity (i.e. increased DA synthesis). The authors reasoned that increased synthesis occurs in response to a disruption of DA neurotransmission leading to abnormally low extracellular levels, as has been shown for the effect of L-DOPA on the dorsal striatum in Parkinson’s disease (150, 151). Presynaptic DA autoreceptor stimulation is thought to play a role in this inverse effect on synthesis/tracer influx (152). The finding of decreased extracellular DA in ADHD has since been replicated through increased prefrontal [^18^F]DOPA influx (153) and increased striatal DAT binding (154–157).

There have, however, been several PET/SPECT studies over the years which have reported findings which either fail to support or are contradictory to the hypo-dopaminergic hypothesis. Studies have found no differences in endogenous DA between ADHD and healthy controls within striatal (158–161) and midbrain regions (160). Of particular interest, a 1998 study be Ernst et al. (162) reported *lower* [^18^F]DOPA uptake in ADHD as opposed to the nonsignificant higher uptake in their 1999 study, by extension implying *increased* extracellular DA in prefrontal regions. Similarly, increased extracellular DA (i.e. lower [^18^F]DOPA influx/DAT binding/receptor binding) has been reported in the striatum (153, 163, 164) and midbrain (153, 158, 165) of ADHD patients relative to controls. This collection of studies would therefore suggest a generalized hyperdopaminergic state in ADHD. For a recent comprehensive meta-analysis of the inconsistent PET and SPECT findings for adult and adolescent ADHD see (166). It may be possible to reconcile the hyper- vs. hypo-dopaminergic findings by considering dynamic versus generalized DA activity. For example, in a task-based study, Badgaiyan and colleagues reported decreased tonic but enhanced phasic release of DA in the right caudate of people with ADHD (167).

In addition to contradictory experimental findings, result interpretations also differ between studies. For example, some authors interpret lower DA synthesis and lower DAT density as evidence of reduced DA signaling and therefore a hypo-functioning dopaminergic system (165), as opposed to the interpretation by Ernst and colleagues in 1999. This discrepancy between interpretations is somewhat unsurprising as translating DA metabolism findings into functional changes in dopaminergic networks can be difficult, given that DA can have both facilitatory and inhibitory effects depending on the dominant local DA receptor population. Nevertheless, the opposing directions for changes in PET/SPECT measures demonstrate incongruent links between ADHD and DA. Possible reasons for these discrepancies include small sample sizes, level of psychostimulant exposure, focus on several kinds of existing ligands, and a questionable validity of the diagnosis through a lack of biological diagnostic criteria (168). The review by Yamamoto and Inada (168) suggests that whether alterations of monoamine function may be involved in the pathophysiological mechanism of adult ADHD remains to be clarified. Indeed, other recent reviews (169) have concluded that there seems to be a greater consensus on other potential disease mechanisms e.g. prefrontal hypo-connectivity and prefrontal glucose hypo-metabolism.

### A role for neurovascular coupling?

As mentioned, all clinically approved stimulant drugs for ADHD either directly or indirectly affect multiple transmitter systems in addition to DA, most notably NE and serotonin neurotransmission. Ultrastructural investigation of DA and NE innervation of cortical structures has revealed a similar morphology of their terminals. Of note, some clinically effective non-stimulant drugs specifically target NE neurotransmission, apparently without directly affecting DA transmission (170). NE is an important modulator of local blood flow in the brain. The clinical efficacy of these medications may imply that the effect of some ADHD medications is not mediated directly on neurons, but rather indirectly *via* effects on blood flow. However, as clinical studies indicate that stimulant drugs that also block the dopamine transporter are more effective than non-stimulant drugs in ADHD, an involvement of DA in their mode of action still seems plausible (13). Nevertheless, such a symptomatic effect of dopaminergic drugs doesn’t necessarily imply that there is an *a priori* defect in the dopamine system.

Neurovascular perfusion is tightly regulated to accommodate changes in neuronal activity and metabolic demand. This neurovascular coupling occurs at the level of the microvasculature – the neurovascular unit – and at larger arteries of the vascular tree. The larger blood vessels are regulated by peripheral innervation, with NE post-ganglionic orthosympathetic fibers and cholinergic parasympathetic fibers. Within the brain parenchyma, precapillary arterioles and capillaries are innervated by NE fibers originating from the locus coeruleus (LC). The brain microvascular compartment is almost completely covered by end-feet projections from nearby astrocytes, leaving a space between the pericyte or smooth muscle layer and the astrocyte end-feet. This space contains the basal matrix of the vasculature and circulating cerebrospinal fluid and is part of the glymphatic system. Most NE varicose release sites target the microvasculature *via* the astrocyte end-feet. Astrocytes have a rich expression of adrenergic receptors, but little is known about expression of specific receptor subtypes.

The underlying signaling mechanisms of neurovascular coupling are still under discussion and the modulatory effect of NE on the functional hyperemia could be context dependent and differ between brain regions. Thus, both vasoconstriction and vasorelaxation have been reported for LC derived NE (171, 172) and a suggested role of NE has been to optimize local blood flow allowing for a focused neurovascular coupling response (171). Very little is known about the neurovascular unit, alterations in neurovascular coupling, and glymphatic flow in ADHD. However, some very recent studies suggest that there could be differences in neurovascular coupling (173) and the glymphatic system (174) in ADHD.

Concluding remarks: Recent PET/SPECT studies do not show predominant evidence supporting a hypo-dopaminergic state in ADHD, but rather conflicting results that imply both increased and decreased extracellular DA levels within frontostriatal regions. The interpretation of PET/SPECT findings are inherently difficult given the level of complexity in dopamine metabolism and signaling as discussed previously, which these modalities struggle to capture with a single ligand-based measure. Functional neuroimaging should be used to further investigate the promising role of neurovascular coupling in ADHD.

## Neuropsychological perspective

To assess the traditional DA theory of ADHD, it is helpful to compare ADHD findings to another neuropsychological condition with a robust and reliable link to deficient DA levels – Parkinson’s disease (PD). Although seemingly on opposite ends of the aging spectrum as a neurodevelopment and neurodegenerative condition, both ADHD and PD are heterogeneous disorders which involve cortico-basal ganglia disturbances that impact the control of motor and cognitive behavior. The root cause of PD symptoms is the degeneration of dopaminergic neurons in the substantia nigra pars compacta (SNpc) which project to the dorsal striatum (**Figure 1**). As such, PD can serve as a useful neurophysiological-behavioral model to reveal the effects of insufficient DA levels in the brain.

The pathological process that underlies PD relentlessly progresses over several years and through specific degenerative stages to the full-blown clinical syndrome (175). PD is classified as a motor disorder and the cardinal symptoms include a pill-rolling tremor at rest, muscular rigidity, bradykinesia and often gait disturbance. The default state of the motor system is analogous to driving with the brakes on. The braking occurs *via* GABAergic input from the basal ganglia (BG) onto thalamocortical neurons (176). Movement initiation is therefore an active process requiring a phasic pause in the tonic inhibition of the thalamus. This is achieved by recruiting the ‘direct’ facilitatory pathway connecting primarily cortical motor areas (i.e. primary motor cortex, supplementary motor area, premotor cortex) straight to the BG output nuclei *via* striatal MSN expressing predominantly D1 receptors. Conversely, recruiting the inhibitory ‘indirect’ pathway originating from striatal neurons expressing predominantly D2 receptors leads to a net increase in thalamic inhibition, and subsequently a decrease in thalamocortical drive. The balance of neuronal output from the BG pathways is usually maintained through nigrostriatal dopaminergic projections. DA binding to DRD1 facilitates the direct pathway, whereas DA binding at DRD2 suppresses the indirect pathway (177, 178). The presence of DA therefore modulates movement control by reinforcing any cortically initiated activation of BG-thalamocortical networks (179). Consequently, decreased nigrostriatal DA results in maladaptive modulation of thalamocortical neurons and a sustained thalamic inhibition (180). This sustained inhibition ultimately presents as a hypo-kinetic state and the paucity of movement common across most cardinal symptoms of PD.

The hypo-active state in PD highlights the apparent contradiction in ADHD that low levels of DA cause motor hyperactivity. However, it may be worth first considering pure motor control in ADHD separately from the motor behaviors resulting from impaired inhibitory control. One could argue that examining pure motor control reveals the influence of nigrostriatal connections in relative isolation. Impaired motor control presents in around 30-50% of children with ADHD (181) but appears to improve with age (182, 183). While reduced frontostriatal activity has repeatedly been found in ADHD including during motor control tasks (184, 185), the source of this dysfunction seems to relate specifically to inhibitory control rather than motor control *per se* (184). Theories of hypo-functioning nigrostriatal connections have been proposed for ADHD (186) which would be expected to produce PD-type motor behaviors. Although some decreases in gross and simple fine motor speed have been reported for individuals with ADHD (183), a similar number of studies have found no differences in fine motor speed, but rather increased variability and reduced movement accuracy (187, 188). Of note, variability and accuracy deficits match those consistently and reliably linked to cerebellar function. Reduced cerebellar volume and activity (189, 190) as well as altered cerebellar neurochemistry (191) have been reported in ADHD. Furthermore, Pitcher et al. (192) found the fine motor control deficits in children with ADHD showed a strong correlation with those in children with dyspraxia. Cerebellar dysfunction could be impacting frontostriatal activity through cortico-cerebellar-thalamo-cortical pathways, specifically the interaction with BG circuitry *via* dentate nucleus output to the striatum (193). The involvement of dysfunctional cerebellar-prefrontal circuitry is not a new idea [e.g (189)]. Although it is worth noting, the consensus is shifting to acknowledge the cerebellum’s role also in non-motor functions. Through cortico-cerebellar-thalamo-prefrontal cortex circuits the cerebellum may play a role in cognitive functions including directed attention (194). Overall, the basic problems of motor control present in ADHD seem very distinct from the slowness of movement caused by deficient nigrostriatal DA in PD and may be due to discrete underlying neural mechanisms.

Abnormal dopaminergic mesocorticolimbic (MCL) pathways are implicated in impaired inhibitory control for both PD (195–197) and ADHD (186, 198, 199). Unlike ADHD, difficulty inhibiting inappropriate motor activity is not a diagnostic criterion for PD but has been found experimentally. It is worth acknowledging that some researchers argue that deficits of inhibitory function in ADHD reflect impairments in basic information processing rather than impulse control (200–202). Nevertheless, both conditions can exhibit impulsive behavior, but again, the underlying mechanisms seem distinct. In early PD, DA concentrations are decreased within the striatum, yet paradoxically DA concentration is increased in the PFC (203). This counterintuitive increase in PFC DA could reflect compensation within the MCL network (204, 205), or/and it could be resulting from reduced striatal DA levels, given the well-studied inverse relationship between MCL and nigrostriatal dopaminergic systems (195, 206–208). Furthermore, the hyperdopaminergic state of the MCL system is believed to be exacerbated by DA medication in some people - especially by DA agonists targeting D2/D3 receptors (209, 210) and lead to full-blown impulse control disorders. Conversely, motor impulsivity/hyperactivity in ADHD is linked to a hypo-dopaminergic (211) and hypo-functional state [for meta-analyses see (212–214)] in the PFC. The hyper- versus hypo-dopaminergic findings between conditions may seem incongruous but could fit with the inverted-U relationship (215) between PFC functions like inhibitory control and DA levels (216, 217) - for a review see Brennan and Arnsten (218). According to this framework, PFC DA concentrations above or below optimal levels will lead to deficits in function. However, the inverted-U is a simplified framework and the relationship between frontostriatal DA and functional performance is of course more complex and includes several contributing factors. Of most relevance to this review, one of the factors thought to influence a person’s position on the inverted-U curve for inhibitory control is DA-related genes (216, 217, 219). Genetic variability will likely have the greatest influence on behavior when DA neurotransmission deviates from close-to-optimal levels (i.e., DA dysregulation). For example, at either extreme end of the inverted-U relationship.

Concluding remarks: Comparing cognitive-motor behaviours resulting from known DA deficiency in PD to those in ADHD reveals several key distinctions, including discrete underlying neural mechanisms.

## Conclusions and future directions

As described in this review, the DA hypothesis of ADHD has been around for several decades and has been formulated in different ways. The current narrative review took a multidisciplinary approach to consider the neuropsychiatric condition in the context of other single gene disorders, metabolic disorders, and neurological disorders with established dysfunction of the dopaminergic system. In this way, the disorders served as models to assess against the different aspects of the DA hypothesis for ADHD. **Table 1** summarizes the main arguments for and against the DA hypothesis from each perspective. Examining the DA hypothesis in this way has produced the following conclusions:

1. Core DA-related genes are not among the major risk genes for ADHD in GWA studies. Compared to GWA studies, candidate gene studies focusing on DA-related gene variants have suffered from low power and inconsistent results. However, an enrichment of ADHD risk variants has been found in genes expressed in excitatory and inhibitory neurons and in midbrain dopaminergic neurons.
2. Humans with rare metabolic diseases with documented DA deficiency have motor neurological symptoms but do not show typical symptoms of ADHD. However, some metabolic diseases that indirectly interfere with DA metabolism show ADHD-like symptoms.
3. There is no consistent neurophysiological support for a lack of frontostriatal DA in ADHD. Evidence exists for a decrease, increase, and no change in extracellular DA in ADHD relative to healthy controls.
4. Parkinson’s disease – as a neurological disorder with well-documented degeneration of DA tracts and a DA deficit – causes a subset of symptoms which overlap somewhat with ADHD. However, the neural mechanisms underlying these behaviors are clearly distinct between the two conditions.

**Table 1:** A brief summary of arguments for and against the dopamine hypothesis for ADHD as detailed from each perspective.

Perspective	Arguments in favor of low dopamine levels as key in ADHD	Arguments against low dopamine levels as key in ADHD
**Genetic:** Animal models	Many animal models with alterations in DA related genes show some ADHD-like behaviors, including hyperactivity and inattention (70, 74).	The phenotypic resemblance of Coloboma mice, SHRs or many other animal models to ADHD cannot be solely attributed to the DA system (17, 70).
**Genetic:** Human molecular genetic studies	Early candidate gene studies for ADHD found altered frequencies of polymorphisms in key genes involved in DA signaling, in particular *DRD4* and *DAT1 (SLC6A3)* (83–89).	Larger replication studies on variants from early candidate gene studies have given inconsistent results (96–98). Recently, large GWA studies have revealed many genes and mechanisms in ADHD, but “core” DA genes are not among the top genes (71, 72, 100).
**Neurometabolic:** Rare Mendelian human diseases with impaired synthesis of brain DA	People with low DA levels in the brain can present with a subset of features that overlap with broader ADHD symptoms, like motor dysfunction and irritability (135, 139).	People with low DA levels exhibit primarily motor neurological symptoms, not cardinal ADHD symptoms (5, 28, 132, 136, 137).
**Other metabolic disorders:** Aminoacidopathies PKU and TYRSN1	There is an increased prevalence of ADHD in patients with metabolic disorders that affect DA production (139–141).	Such neurometabolic disorders can affect multiple biological pathways, not only DA signaling (145–147). Some conditions may manifest without prominent alterations to brain function (148).
**Neurophysiological:** PET and SPECT studies in people with and without ADHD	People with ADHD have higher midbrain and frontal [^18^F]DOPA intake and increased striatal DAT binding, indicating lower extracellular DA tone (149, 153–157).	People with ADHD have lower [^18^F]DOPA intake, decreased DAT binding and decreased D2/3 receptor binding, implying increased extracellular DA tone (153, 158, 162–165). Several studies have found no differences in endogenous DA between ADHD and healthy controls within striatal and midbrain regions, failing to replicate early findings (158–161).
**Neuropsychological:** Using Parkinson’s disease as a model for dysfunctional dopaminergic connections	People with both ADHD and PD exhibit problems with impulse control and changes to motor control (175, 176, 181–183).	Motor deficits are distinct between the conditions with a hypo-active state in PD versus a hyper-active state in ADHD. Opposing mechanisms underly impaired impulse control in ADHD vs PD as a hypo- versus hyper-dopaminergic state in the PFC, respectively (203, 209–214, 218).
Additional evidence discussed		
Pharmacology of stimulant drugs used to treat ADHD	Amphetamines and similar stimulants drugs block monoamine transporters and transiently increase synaptic levels of DA (and NE). Amphetamine treatment may result in cognitive improvements in certain patients with known metabolic syndromes affecting DA synthesis (14, 139–141).	Simulants also affect other neurotransmitter systems. Non-stimulant drugs primarily affecting NE transmission are also effective in ADHD (13). Stimulants have similar effects in most people, irrespective of whether they have ADHD or not (16, 17).

For more detailed arguments see the main text.

The updated overview therefore points to the fact that there is ample evidence for some involvement of DA but limited evidence that reduced levels of the DA neurotransmitter *per se* is a defining feature of ADHD. Based on the variable findings from clinical, genetic, imaging and neurophysiological studies, it is possible that the multiple underlying pathophysiological mechanisms in ADHD are differently involved and that the putative alterations in DA functions are only present in a subset of ADHD patients. Identification of such alterations may be essential to tailor individualized treatments. Although early pharmacogenetic studies on monoaminergic candidate genes have given mixed results (220), this field is rapidly evolving, and pharmacogenomics may be used to optimize treatment selection (i.e. genome guided “precision medicine”) (221).

It appears that important aspects for future work includes dopamine’s role during perinatal brain development and how DA interacts with other monoamines (e.g. NE) and/or neurotransmitters [e.g. GABA (222)]. Considering the close interactions between DA, NE and serotonin neurotransmission, it is difficult to study these systems in isolation. Impaired DA synthesis will always be accompanied by decreased NE production. Pharmacological or genetic blocking of DA related enzymes or receptors results in secondary and compensatory alterations in receptor densities and other signaling components in these and other transmitter systems. Although not the focus of this review, there is also emerging evidence that ADHD is associated with white matter disruptions to multiple cortical pathways (223).

It is perhaps unsurprising that ADHD as an umbrella term encompassing a range of complex behavioral changes should also be underpinned by intricate neurophysiological changes unable to be adequately explained by any single neurotransmitter. Indeed, the 2021 international consensus statement from the World Federation of ADHD who curated findings with a strong evidence base to generate 208 empirically supported statements about ADHD, did not specifically single-out DA as a key neurotransmitter for the condition (224). Part of their conclusion was that: “There are multiple genetic and environmental risk factors that accumulate in various combinations to cause ADHD. These risk factors lead to subtle changes in multiple brain networks and in the cognitive, motivational, and emotional processes they control.”

The extent of conflicting findings in human neuroimaging studies is somewhat surprising. There are clearly advantages to being able to probe dopaminergic function *in vivo* with PET and SPECT. However, there are also practical and theoretical limitations to these neuroimaging techniques that should be considered when interpreting the body of literature. Practically, there are always potential problems with artifacts during attenuation correction which can cause inaccurate estimations of radiotracer uptake and/or incorrect positioning of uptake sites (225). Theoretically, as mentioned, the interpretation of PET/SPECT findings are inherently difficult given the level of complexity in dopamine metabolism alluded to in this review, which is impossible to capture with a single radiotracer-based measure. Translating DA metabolism findings into functional changes in dopaminergic networks is challenging, given that DA can have both facilitatory and inhibitory effects depending on the dominant local DA receptor population. Therefore, using PET/SPECT as one piece of a multidisciplinary puzzle might be required to tease apart the variability in neuroimaging findings and reveal a consistent dopaminergic state in ADHD.

### Emerging perspectives

The aim of the current review was to assess ADHD from a multidisciplinary perspective. However, we are of course unable to consider the condition from all perspectives, and our review of the literature has also been limited to established fields with larger bodies of research into ADHD. As multi-omics, imaging and computational studies are making fast progress, there are other emerging avenues of investigation which will contribute to the developing picture of dopaminergic (dys)function in ADHD as presented in this review. For example, neurocomputational modeling of impaired presynaptic DA regulation (226) allows the simulation of decreased tonic and simultaneously increased phasic frontostriatal dopaminergic activity. Such methods could perhaps help to reconcile the hyper- vs. hypo-dopaminergic findings from neuroimaging studies. As briefly discussed, the role of neurovascular coupling in ADHD also appears to be an emerging idea that warrants further investigation utilizing the accelerating capabilities of functional neuroimaging methods. Increasing evidence also supports a role for the gut microbiome in human health and behavior, including ADHD (227). Therefore, the gut microbiome will be an important part of future multi-omics investigations. Overall, the diversity of promising research areas highlights the potential for integrative explorations into ADHD from an even more comprehensive multidisciplinary perspective, which will most likely be needed to truly understand the complex neurobiology of this condition.

## References

[1] Fahn S. The history of dopamine and levodopa in the treatment of Parkinson's disease. Mov Disord. (2008) 23 Suppl 3:S497–508. doi: 10.1002/mds.22028 18781671

[2] Montagu KA. Catechol compounds in rat tissues and in brains of different animals. Nature. (1957) 180:244–5. doi: 10.1038/180244a0 13451690

[3] Benes FM. Carlsson and the discovery of dopamine. Trends Pharmacol Sci. (2001) 22:46–7. doi: 10.1016/S0165-6147(00)01607-2 11165672

[4] Hornykiewicz O. The discovery of dopamine deficiency in the parkinsonian brain. J Neural Transm Suppl. (2006) 70:9–15. doi: 10.1007/978-3-211-45295-0_3 17017502

[5] Nygaard G Szigetvari PD Grindheim AK Ruoff P Martinez A Haavik J. Personalized medicine to improve treatment of dopa-responsive dystonia-A focus on tyrosine hydroxylase deficiency. J Pers Med. (2021) 11. doi: 10.3390/jpm11111186 PMC8625014 34834538

[6] American Psychiatric Association. Diagnostic and Statistical Manual of Mental Disorders. 5th ed. Washington, DC: American Psychiatric Publishing (2013).

[7] Wender PH. Some speculations concerning a possible biochemical basis of minimal brain dysfunction. Ann N Y Acad Sci. (1973) 205:18–28. doi: 10.1111/j.1749-6632.1973.tb43159.x 4570233

[8] Shaywitz SE Cohen DJ Shaywitz BA. The biochemical basis of minimal brain dysfunction. J Pediatr. (1978) 92:179–87. doi: 10.1016/S0022-3476(78)80001-8 340625

[9] Kollins SH Adcock RA. ADHD, altered dopamine neurotransmission, and disrupted reinforcement processes: implications for smoking and nicotine dependence. Prog Neuropsychopharmacol Biol Psychiatry. (2014) 52:70–8. doi: 10.1016/j.pnpbp.2014.02.002 PMC4004668 24560930

[10] Hallowell EM Ratey JJ. Driven to Distraction: Recognizing and Coping with Attention Deficit Disorder. Anchor: Vintage (2011).

[11] Yeung A Ng E Abi-Jaoude E. TikTok and attention-deficit/hyperactivity disorder: A cross-sectional study of social media content quality. Can J Psychiatry. (2022) 67:899–906. doi: 10.1177/07067437221082854 35196157 PMC9659797

[12] Zametkin AJ Rapoport JL. Neurobiology of attention deficit disorder with hyperactivity: where have we come in 50 years? J Am Acad Child Adolesc Psychiatry. (1987) 26:676–86. doi: 10.1097/00004583-198709000-00011 2889717

[13] Cortese S. Pharmacologic treatment of attention deficit-hyperactivity disorder. N Engl J Med. (2020) 383:1050–6. doi: 10.1056/NEJMra1917069 32905677

[14] Faraone SV. The pharmacology of amphetamine and methylphenidate: Relevance to the neurobiology of attention-deficit/hyperactivity disorder and other psychiatric comorbidities. Neurosci Biobehav Rev. (2018) 87:255–70. doi: 10.1016/j.neubiorev.2018.02.001 PMC8063758 29428394

[15] Parlatini V Bellato A Murphy D Cortese S. From neurons to brain networks, pharmacodynamics of stimulant medication for ADHD. Neurosci Biobehav Rev. (2024) 164:105841. doi: 10.1016/j.neubiorev.2024.105841 39098738

[16] Pliszka SR. The neuropsychopharmacology of attention-deficit/hyperactivity disorder. Biol Psychiatry. (2005) 57:1385–90. doi: 10.1016/j.biopsych.2004.08.026 15950012

[17] Gonon F. The dopaminergic hypothesis of attention-deficit/hyperactivity disorder needs re-examining. Trends Neurosci. (2009) 32:2–8. doi: 10.1016/j.tins.2008.09.010 18986716

[18] Rapoport JL Buchsbaum MS Zahn TP Weingartner H Ludlow C Mikkelsen EJ. Dextroamphetamine: cognitive and behavioral effects in normal prepubertal boys. Science. (1978) 199:560–3. doi: 10.1126/science.341313 341313

[19] Rapoport JL Inoff-Germain G. Responses to methylphenidate in Attention-Deficit/Hyperactivity Disorder and normal children: update 2002. J Atten Disord. (2002) 6 Suppl 1:S57–60. doi: 10.1177/070674370200601S07 12685519

[20] van der Kooij MA Glennon JC. Animal models concerning the role of dopamine in attention-deficit hyperactivity disorder. Neurosci Biobehav Rev. (2007) 31:597–618. doi: 10.1016/j.neubiorev.2006.12.002 17316796

[21] Dickstein SG Bannon K Castellanos FX Milham MP. The neural correlates of attention deficit hyperactivity disorder: an ALE meta-analysis. J Child Psychol Psychiatry. (2006) 47:1051–62. doi: 10.1111/j.1469-7610.2006.01671.x 17073984

[22] Isaac V Lopez V Escobar MJ. Arousal dysregulation and executive dysfunction in attention deficit hyperactivity disorder (ADHD). Front Psychiatry. (2023) 14:1336040. doi: 10.3389/fpsyt.2023.1336040 38298926 PMC10827919

[23] Senkowski D Ziegler T Singh M Heinz A He J Silk T. Assessing inhibitory control deficits in adult ADHD: A systematic review and meta-analysis of the stop-signal task. Neuropsychol Rev. (2024) 34:548–67. doi: 10.1007/s11065-023-09592-5 PMC11166755 37300725

[24] Levy F. The dopamine theory of attention deficit hyperactivity disorder (ADHD). Aust N Z J Psychiatry. (1991) 25:277–83. doi: 10.3109/00048679109077746 1652243

[25] Klein MO Battagello DS Cardoso AR Hauser DN Bittencourt JC Correa RG. Dopamine: functions, signaling, and association with neurological diseases. Cell Mol Neurobiol. (2019) 39:31–59. doi: 10.1007/s10571-018-0632-3 30446950 PMC11469830

[26] Jin R Sun S Hu Y Zhang H Sun X. Neuropeptides modulate feeding via the dopamine reward pathway. Neurochem Res. (2023) 48:2622–43. doi: 10.1007/s11064-023-03954-4 37233918

[27] Matt SM Gaskill PJ. Where Is Dopamine and how do Immune Cells See it?: Dopamine-Mediated Immune Cell Function in Health and Disease. J Neuroimmune Pharmacol. (2020) 15:114–64. doi: 10.1007/s11481-019-09851-4 PMC6842680 31077015

[28] Haavik J Toska K. Tyrosine hydroxylase and Parkinson's disease. Mol Neurobiol. (1998) 16:285–309. doi: 10.1007/BF02741387 9626667

[29] Meiser J Weindl D Hiller K. Complexity of dopamine metabolism. Cell Commun Signal. (2013) 11:34. doi: 10.1186/1478-811X-11-34 23683503 PMC3693914

[30] Liu C Goel P Kaeser PS. Spatial and temporal scales of dopamine transmission. Nat Rev Neurosci. (2021) 22:345–58. doi: 10.1038/s41583-021-00455-7 PMC8220193 33837376

[31] Cragg SJ Greenfield SA. Differential autoreceptor control of somatodendritic and axon terminal dopamine release in substantia nigra, ventral tegmental area, and striatum. J Neurosci. (1997) 17:5738–46. doi: 10.1523/JNEUROSCI.17-15-05738.1997 PMC6573186 9221772

[32] Ciliax BJ Drash GW Staley JK Haber S Mobley CJ Miller GW. Immunocytochemical localization of the dopamine transporter in human brain. J Comp Neurol. (1999) 409:38–56. doi: 10.1002/(SICI)1096-9861(19990621)409:1<38::AID-CNE4>3.0.CO;2-1 10363710

[33] Beaulieu JM Gainetdinov RR. The physiology, signaling, and pharmacology of dopamine receptors. Pharmacol Rev. (2011) 63:182–217. doi: 10.1124/pr.110.002642 21303898

[34] Xu P Huang S Krumm BE Zhuang Y Mao C Zhang Y. Structural genomics of the human dopamine receptor system. Cell Res. (2023) 33:604–16. doi: 10.1038/s41422-023-00808-0 PMC10397222 37221270

[35] Felder CC Jose PA Axelrod J. The dopamine-1 agonist, SKF 82526, stimulates phospholipase-C activity independent of adenylate cyclase. J Pharmacol Exp Ther. (1989) 248:171–5. 2563286

[36] Beaulieu JM Espinoza S Gainetdinov RR. Dopamine receptors - IUPHAR review 13. Br J Pharmacol. (2015) 172:1–23. doi: 10.1111/bph.2015.172.issue-1 25671228 PMC4280963

[37] Perreault ML Hasbi A O'Dowd BF George SR. Heteromeric dopamine receptor signaling complexes: emerging neurobiology and disease relevance. Neuropsychopharmacology. (2014) 39:156–68. doi: 10.1038/npp.2013.148 PMC3857642 23774533

[38] Benac N Ezequiel Saraceno G Butler C Kuga N Nishimura Y Yokoi T. Non-canonical interplay between glutamatergic NMDA and dopamine receptors shapes synaptogenesis. Nat Commun. (2024) 15:27. doi: 10.1038/s41467-023-44301-z 38167277 PMC10762086

[39] Homar-Ruano P Cai NS Casado-Anguera V Casado V Ferre S Moreno E. Significant functional differences between dopamine D(4) receptor polymorphic variants upon heteromerization with alpha(1A) adrenoreceptors. Mol Neurobiol. (2023) 60:6566–83. doi: 10.1007/s12035-023-03476-8 PMC10533593 37464153

[40] Frederick AL Yano H Trifilieff P Vishwasrao HD Biezonski D Meszaros J. Evidence against dopamine D1/D2 receptor heteromers. Mol Psychiatry. (2015) 20:1373–85. doi: 10.1038/mp.2014.166 PMC4492915 25560761

[41] Speranza L di Porzio U Viggiano D de Donato A Volpicelli F. Dopamine: the neuromodulator of long-term synaptic plasticity, reward and movement control. Cells. (2021) 10. doi: 10.3390/cells10040735 PMC8066851 33810328

[42] Maurice N Tkatch T Meisler M Sprunger LK Surmeier DJ. D1/D5 dopamine receptor activation differentially modulates rapidly inactivating and persistent sodium currents in prefrontal cortex pyramidal neurons. J Neurosci. (2001) 21:2268–77. doi: 10.1523/JNEUROSCI.21-07-02268.2001 PMC6762404 11264302

[43] Gong S Fayette N Heinsbroek JA Ford CP. Cocaine shifts dopamine D2 receptor sensitivity to gate conditioned behaviors. Neuron. (2022) 110:1272. doi: 10.1016/j.neuron.2022.03.005 35390291 PMC9017772

[44] Ford CP. The role of D2-autoreceptors in regulating dopamine neuron activity and transmission. Neuroscience. (2014) 282:13–22. doi: 10.1016/j.neuroscience.2014.01.025 24463000 PMC4108583

[45] Ijomone OK Oria RS Ijomone OM Aschner M Bornhorst J. Dopaminergic perturbation in the aetiology of neurodevelopmental disorders. Mol Neurobiol. (2024). doi: 10.1007/s12035-024-04418-8 PMC11772124 39110391

[46] Zhang YQ Lin WP Huang LP Zhao B Zhang CC Yin DM. Dopamine D2 receptor regulates cortical synaptic pruning in rodents. Nat Commun. (2021) 12:6444. doi: 10.1038/s41467-021-26769-9 34750364 PMC8576001

[47] Zhuo M. Neural mechanisms underlying anxiety-chronic pain interactions. Trends Neurosci. (2016) 39:136–45. doi: 10.1016/j.tins.2016.01.006 26878750

[48] Pramod AB Foster J Carvelli L Henry LK. SLC6 transporters: structure, function, regulation, disease association and therapeutics. Mol Aspects Med. (2013) 34:197–219. doi: 10.1016/j.mam.2012.07.002 23506866 PMC3602807

[49] Srivastava DK Navratna V Tosh DK Chinn A Sk MF Tajkhorshid E. Structure of the human dopamine transporter and mechanisms of inhibition. Nature. (2024) 632:672–7. doi: 10.1038/s41586-024-07739-9 PMC11324517 39112705

[50] Wang Y Zhang P Chao Y Zhu Z Yang C Zhou Z. Transport and inhibition mechanism for VMAT2-mediated synaptic vesicle loading of monoamines. Cell Res. (2024) 34:47–57. doi: 10.1038/s41422-023-00906-z 38163846 PMC10770148

[51] Bernstein AI Stout KA Miller GW. The vesicular monoamine transporter 2: an underexplored pharmacological target. Neurochem Int. (2014) 73:89–97. doi: 10.1016/j.neuint.2013.12.003 24398404 PMC5028832

[52] Chen J Song J Yuan P Tian Q Ji Y Ren-Patterson R. Orientation and cellular distribution of membrane-bound catechol-O-methyltransferase in cortical neurons: implications for drug development. J Biol Chem. (2011) 286:34752–60. doi: 10.1074/jbc.M111.262790 PMC3186432 21846718

[53] Dickson PW Briggs GD. Tyrosine hydroxylase: regulation by feedback inhibition and phosphorylation. Adv Pharmacol. (2013) 68:13–21. doi: 10.1016/B978-0-12-411512-5.00002-6 24054138

[54] Rostrup M Fossbakk A Hauge A Kleppe R Gnaiger E Haavik J. Oxygen dependence of tyrosine hydroxylase. Amino Acids. (2008) 34:455–64. doi: 10.1007/s00726-007-0547-7 17520326

[55] Pardridge WM. Blood-brain barrier carrier-mediated transport and brain metabolism of amino acids. Neurochem Res. (1998) 23:635–44. doi: 10.1023/A:1022482604276 9566601

[56] Szigetvari PD Patil S Birkeland E Kleppe R Haavik J. The effects of phenylalanine and tyrosine levels on dopamine production in rat PC12 cells. Implications for treatment of phenylketonuria, tyrosinemia type 1 and comorbid neurodevelopmental disorders. Neurochem Int. (2023) 171:105629. doi: 10.1016/j.neuint.2023.105629 37865339

[57] Dunkley PR Dickson PW. Tyrosine hydroxylase phosphorylation in vivo. J Neurochem. (2019) 149:706–28. doi: 10.1111/jnc.2019.149.issue-6 30714137

[58] Lindgren N Xu ZQ Lindskog M Herrera-Marschitz M Goiny M Haycock J. Regulation of tyrosine hydroxylase activity and phosphorylation at Ser(19) and Ser(40) via activation of glutamate NMDA receptors in rat striatum. J Neurochem. (2000) 74:2470–7. doi: 10.1046/j.1471-4159.2000.0742470.x 10820208

[59] Lindgren N Goiny M Herrera-Marschitz M Haycock JW Hokfelt T Fisone G. Activation of extracellular signal-regulated kinases 1 and 2 by depolarization stimulates tyrosine hydroxylase phosphorylation and dopamine synthesis in rat brain. Eur J Neurosci. (2002) 15:769–73. doi: 10.1046/j.1460-9568.2002.01901.x 11886455

[60] Ghorbani S Szigetvari PD Haavik J Kleppe R. Serine 19 phosphorylation and 14-3-3 binding regulate phosphorylation and dephosphorylation of tyrosine hydroxylase on serine 31 and serine 40. J Neurochem. (2020) 152:29–47. doi: 10.1111/jnc.v152.1 31529487

[61] Duchemin AM Berry MD Neff NH Hadjiconstantinou M. Phosphorylation and activation of brain aromatic L-amino acid decarboxylase by cyclic AMP-dependent protein kinase. J Neurochem. (2000) 75:725–31. doi: 10.1046/j.1471-4159.2000.0750725.x 10899948

[62] German CL Baladi MG McFadden LM Hanson GR Fleckenstein AE. Regulation of the dopamine and vesicular monoamine transporters: pharmacological targets and implications for disease. Pharmacol Rev. (2015) 67:1005–24. doi: 10.1124/pr.114.010397 PMC4630566 26408528

[63] Menniti FS Faraci WS Schmidt CJ. Phosphodiesterases in the CNS: targets for drug development. Nat Rev Drug Discovery. (2006) 5:660–70. doi: 10.1038/nrd2058 16883304

[64] Lakics V Karran EH Boess FG. Quantitative comparison of phosphodiesterase mRNA distribution in human brain and peripheral tissues. Neuropharmacology. (2010) 59:367–74. doi: 10.1016/j.neuropharm.2010.05.004 20493887

[65] Delhaye S Bardoni B. Role of phosphodiesterases in the pathophysiology of neurodevelopmental disorders. Mol Psychiatry. (2021) 26:4570–82. doi: 10.1038/s41380-020-00997-9 PMC8589663 33414502

[66] Xu Y Zhang HT O'Donnell JM. Phosphodiesterases in the central nervous system: implications in mood and cognitive disorders. Handb Exp Pharmacol. (2011) 204:447–85. doi: 10.1007/978-3-642-17969-3_19 21695652

[67] Mota E Bompierre S Betolngar D Castro LRV Vincent P. Pivotal role of phosphodiesterase 10A in the integration of dopamine signals in mice striatal D(1) and D(2) medium-sized spiny neurones. Br J Pharmacol. (2021) 178:4873–90. doi: 10.1111/bph.v178.24 34399440

[68] Stephenson DT Coskran TM Kelly MP Kleiman RJ Morton D O'Neill SM. The distribution of phosphodiesterase 2A in the rat brain. Neuroscience. (2012) 226:145–55. doi: 10.1016/j.neuroscience.2012.09.011 PMC4409981 23000621

[69] Nishi A Kuroiwa M Miller DB O'Callaghan JP Bateup HS Shuto T. Distinct roles of PDE4 and PDE10A in the regulation of cAMP/PKA signaling in the striatum. J Neurosci. (2008) 28:10460–71. doi: 10.1523/JNEUROSCI.2518-08.2008 PMC2814340 18923023

[70] Cabana-Dominguez J Anton-Galindo E Fernandez-Castillo N Singgih EL O'Leary A Norton WH. The translational genetics of ADHD and related phenotypes in model organisms. Neurosci Biobehav Rev. (2023) 144:104949. doi: 10.1016/j.neubiorev.2022.104949 36368527

[71] Demontis D Walters RK Martin J Mattheisen M Als TD Agerbo E. Discovery of the first genome-wide significant risk loci for attention deficit/hyperactivity disorder. Nat Genet. (2019) 51:63–75. doi: 10.1038/s41588-018-0269-7 30478444 PMC6481311

[72] Demontis D Walters GB Athanasiadis G Walters R Therrien K Nielsen TT. Genome-wide analyses of ADHD identify 27 risk loci, refine the genetic architecture and implicate several cognitive domains. Nat Genet. (2023) 55:198–208. doi: 10.1038/s41588-022-01285-8 36702997 PMC10914347

[73] Ahmed NI Khandelwal N Anderson AG Oh E Vollmer RM Kulkarni A. Compensation between FOXP transcription factors maintains proper striatal function. Cell Rep. (2024) 43:114257. doi: 10.1016/j.celrep.2024.114257 38761373 PMC11234887

[74] Viggiano D Ruocco LA Arcieri S Sadile AG. Involvement of norepinephrine in the control of activity and attentive processes in animal models of attention deficit hyperactivity disorder. Neural Plast. (2004) 11:133–49. doi: 10.1155/NP.2004.133 PMC2565437 15303310

[75] Giros B Jaber M Jones SR Wightman RM Caron MG. Hyperlocomotion and indifference to cocaine and amphetamine in mice lacking the dopamine transporter. Nature. (1996) 379:606–12. doi: 10.1038/379606a0 8628395

[76] Rocha BA Fumagalli F Gainetdinov RR Jones SR Ator R Giros B. Cocaine self-administration in dopamine-transporter knockout mice. Nat Neurosci. (1998) 1:132–7. doi: 10.1038/381 10195128

[77] Harris SS Green SM Kumar M Urs NM. A role for cortical dopamine in the paradoxical calming effects of psychostimulants. Sci Rep. (2022) 12:3129. doi: 10.1038/s41598-022-07029-2 35210489 PMC8873208

[78] Sora I Hall FS Andrews AM Itokawa M Li XF Wei HB. Molecular mechanisms of cocaine reward: combined dopamine and serotonin transporter knockouts eliminate cocaine place preference. Proc Natl Acad Sci U S A. (2001) 98:5300–5. doi: 10.1073/pnas.091039298 PMC33204 11320258

[79] Larsson H Chang Z D'Onofrio BM Lichtenstein P. The heritability of clinically diagnosed attention deficit hyperactivity disorder across the lifespan. Psychol Med. (2014) 44:2223–9. doi: 10.1017/S0033291713002493 PMC4071160 24107258

[80] Faraone SV Larsson H. Genetics of attention deficit hyperactivity disorder. Mol Psychiatry. (2019) 24:562–75. doi: 10.1038/s41380-018-0070-0 PMC6477889 29892054

[81] Franke B Faraone SV Asherson P Buitelaar J Bau CH Ramos-Quiroga JA. The genetics of attention deficit/hyperactivity disorder in adults, a review. Mol Psychiatry. (2012) 17:960–87. doi: 10.1038/mp.2011.138 PMC3449233 22105624

[82] Franke B Michelini G Asherson P Banaschewski T Bilbow A Buitelaar JK. Live fast, die young? A review on the developmental trajectories of ADHD across the lifespan. Eur Neuropsychopharmacol. (2018) 28:1059–88. doi: 10.1016/j.euroneuro.2018.08.001 PMC6379245 30195575

[83] Wallis D Russell HF Muenke M. Review: Genetics of attention deficit/hyperactivity disorder. J Pediatr Psychol. (2008) 33:1085–99. doi: 10.1093/jpepsy/jsn049 18522996

[84] Brookes K Xu X Chen W Zhou K Neale B Lowe N. The analysis of 51 genes in DSM-IV combined type attention deficit hyperactivity disorder: association signals in DRD4, DAT1 and 16 other genes. Mol Psychiatry. (2006) 11:934–53. doi: 10.1038/sj.mp.4001869 16894395

[85] Johansson S Halleland H Halmoy A Jacobsen KK Landaas ET Dramsdahl M. Genetic analyses of dopamine related genes in adult ADHD patients suggest an association with the DRD5-microsatellite repeat, but not with DRD4 or SLC6A3 VNTRs. Am J Med Genet B Neuropsychiatr Genet. (2008) 147B:1470–5. doi: 10.1002/ajmg.b.v147b:8 18081165

[86] Franke B Vasquez AA Johansson S Hoogman M Romanos J Boreatti-Hummer A. Multicenter analysis of the SLC6A3/DAT1 VNTR haplotype in persistent ADHD suggests differential involvement of the gene in childhood and persistent ADHD. Neuropsychopharmacology. (2010) 35:656–64. doi: 10.1038/npp.2009.170 PMC3055604 19890261

[87] Landaas ET Johansson S Jacobsen KK Ribases M Bosch R Sanchez-Mora C. An international multicenter association study of the serotonin transporter gene in persistent ADHD. Genes Brain Behav. (2010) 9:449–58. doi: 10.1111/j.1601-183X.2010.00567.x 20113357

[88] Gizer IR Ficks C Waldman ID. Candidate gene studies of ADHD: a meta-analytic review. Hum Genet. (2009) 126:51–90. doi: 10.1007/s00439-009-0694-x 19506906

[89] Bonvicini C Faraone SV Scassellati C. Common and specific genes and peripheral biomarkers in children and adults with attention-deficit/hyperactivity disorder. World J Biol Psychiatry. (2018) 19:80–100. doi: 10.1080/15622975.2017.1282175 28097908 PMC5568996

[90] Lichter JB Barr CL Kennedy JL Van Tol HH Kidd KK Livak KJ. A hypervariable segment in the human dopamine receptor D4 (DRD4) gene. Hum Mol Genet. (1993) 2:767–73. doi: 10.1093/hmg/2.6.767 8353495

[91] Ding YC Chi HC Grady DL Morishima A Kidd JR Kidd KK. Evidence of positive selection acting at the human dopamine receptor D4 gene locus. Proc Natl Acad Sci U S A. (2002) 99:309–14. doi: 10.1073/pnas.012464099 PMC117557 11756666

[92] Naka I Nishida N Ohashi J. No evidence for strong recent positive selection favoring the 7 repeat allele of VNTR in the DRD4 gene. PLoS One. (2011) 6:e24410. doi: 10.1371/journal.pone.0024410 21909391 PMC3164202

[93] Taub DR Page J. Molecular signatures of natural selection for polymorphic genes of the human dopaminergic and serotonergic systems: A review. Front Psychol. (2016) 7:857. doi: 10.3389/fpsyg.2016.00857 27375535 PMC4896960

[94] Baum L Lee CC Ye R Zhong Y Hung SF Tang CP. Attention-deficit/hyperactivity disorder and dopamine receptor D4 (DRD4) exon 3 variable number of tandem repeats (VNTR) 2-repeat allele. Ann Hum Genet. (2024) 88:382–91. doi: 10.1111/ahg.12560 38624263

[95] Bonvicini C Cortese S Maj C Baune BT Faraone SV Scassellati C. DRD4 48 bp multiallelic variants as age-population-specific biomarkers in attention-deficit/hyperactivity disorder. Transl Psychiatry. (2020) 10:70. doi: 10.1038/s41398-020-0755-4 32075956 PMC7031506

[96] Kirley A Hawi Z Daly G McCarron M Mullins C Millar N. Dopaminergic system genes in ADHD: toward a biological hypothesis. Neuropsychopharmacology. (2002) 27:607–19. doi: 10.1016/S0893-133X(02)00315-9 12377397

[97] Cook EH Jr. Stein MA Krasowski MD Cox NJ Olkon DM Kieffer JE. Association of attention-deficit disorder and the dopamine transporter gene. Am J Hum Genet. (1995) 56:993–8. PMC1801209 7717410

[98] Xu X Mill J Sun B Chen CK Huang YS Wu YY. Association study of promoter polymorphisms at the dopamine transporter gene in Attention Deficit Hyperactivity Disorder. BMC Psychiatry. (2009) 9:3. doi: 10.1186/1471-244X-9-3 19196467 PMC2644291

[99] Grunblatt E Werling AM Roth A Romanos M Walitza S. Association study and a systematic meta-analysis of the VNTR polymorphism in the 3'-UTR of dopamine transporter gene and attention-deficit hyperactivity disorder. J Neural Transm (Vienna). (2019) 126:517–29. doi: 10.1007/s00702-019-01998-x PMC6456487 30923918

[100] Hegvik TA Waloen K Pandey SK Faraone SV Haavik J Zayats T. Druggable genome in attention deficit/hyperactivity disorder and its co-morbid conditions. New avenues for treatment. Mol Psychiatry. (2019) 26:4004–4015. doi: 10.1038/s41380-019-0540-z PMC7165040 31628418

[101] Saunders GRB Wang X Chen F Jang SK Liu M Wang C. Genetic diversity fuels gene discovery for tobacco and alcohol use. Nature. (2022) 612:720–4. doi: 10.1038/s41586-022-05477-4 PMC9771818 36477530

[102] Kimbrel NA Ashley-Koch AE Qin XJ Lindquist JH Garrett ME Dennis MF. Identification of novel, replicable genetic risk loci for suicidal thoughts and behaviors among US military veterans. JAMA Psychiatry. (2023) 80:135–45. doi: 10.1001/jamapsychiatry.2022.3896 PMC9857322 36515925

[103] Karlsson Linner R Biroli P Kong E Meddens SFW Wedow R Fontana MA. Genome-wide association analyses of risk tolerance and risky behaviors in over 1 million individuals identify hundreds of loci and shared genetic influences. Nat Genet. (2019) 51:245–57. doi: 10.1038/s41588-018-0309-3 PMC6713272 30643258

[104] Schizophrenia Working Group of the Psychiatric Genomics C. Biological insights from 108 schizophrenia-associated genetic loci. Nature. (2014) 511:421–7. doi: 10.1038/nature13595 PMC4112379 25056061

[105] Giannakopoulou O Lin K Meng X Su MH Kuo PH Peterson RE. The genetic architecture of depression in individuals of east asian ancestry: A genome-wide association study. JAMA Psychiatry. (2021) 78:1258–69. doi: 10.1001/jamapsychiatry.2021.2099 PMC8482304 34586374

[106] Docherty AR Mullins N Ashley-Koch AE Qin X Coleman JRI Shabalin A. GWAS meta-analysis of suicide attempt: identification of 12 genome-wide significant loci and implication of genetic risks for specific health factors. Am J Psychiatry. (2023) 180:723–38. doi: 10.1176/appi.ajp.21121266 PMC10603363 37777856

[107] Nagel M Jansen PR Stringer S Watanabe K de Leeuw CA Bryois J. Meta-analysis of genome-wide association studies for neuroticism in 449,484 individuals identifies novel genetic loci and pathways. Nat Genet. (2018) 50:920–7. doi: 10.1038/s41588-018-0151-7 29942085

[108] Okbay A Wu Y Wang N Jayashankar H Bennett M Nehzati SM. Polygenic prediction of educational attainment within and between families from genome-wide association analyses in 3 million individuals. Nat Genet. (2022) 54:437–49. doi: 10.1038/s41588-022-01016-z PMC9005349 35361970

[109] Cross-Disorder Group of the Psychiatric Genomics Consortium. Electronic address pmhe Cross-Disorder Group of the Psychiatric Genomics C. Genomic relationships, novel loci, and pleiotropic mechanisms across eight psychiatric disorders. Cell. (2019) 179:1469–82.e11. doi: 10.1016/j.cell.2019.11.020 31835028 PMC7077032

[110] Nagel M Watanabe K Stringer S Posthuma D van der Sluis S. Item-level analyses reveal genetic heterogeneity in neuroticism. Nat Commun. (2018) 9:905. doi: 10.1038/s41467-018-03242-8 29500382 PMC5834468

[111] Rajagopal VM Duan J Vilar-Ribo L Grove J Zayats T Ramos-Quiroga JA. Differences in the genetic architecture of common and rare variants in childhood, persistent and late-diagnosed attention-deficit hyperactivity disorder. Nat Genet. (2022) 54:1117–24. doi: 10.1038/s41588-022-01143-7 PMC10028590 35927488

[112] de Leeuw CA Mooij JM Heskes T Posthuma D. MAGMA: generalized gene-set analysis of GWAS data. PLoS Comput Biol. (2015) 11:e1004219. doi: 10.1371/journal.pcbi.1004219 25885710 PMC4401657

[113] de Leeuw CA Neale BM Heskes T Posthuma D. The statistical properties of gene-set analysis. Nat Rev Genet. (2016) 17:353–64. doi: 10.1038/nrg.2016.29 27070863

[114] DiMaio S Grizenko N Joober R. Dopamine genes and attention-deficit hyperactivity disorder: a review. J Psychiatry Neurosci. (2003) 28:27–38. 12587848 PMC161723

[115] Cabana-Dominguez J Torrico B Reif A Fernandez-Castillo N Cormand B. Comprehensive exploration of the genetic contribution of the dopaminergic and serotonergic pathways to psychiatric disorders. Transl Psychiatry. (2022) 12:11. doi: 10.1038/s41398-021-01771-3 35013130 PMC8748838

[116] Halmoy A Klungsoyr K Skjaerven R Haavik J. Pre- and perinatal risk factors in adults with attention-deficit/hyperactivity disorder. Biol Psychiatry. (2012) 71:474–81. doi: 10.1016/j.biopsych.2011.11.013 22200325

[117] Bala JJ Bala JD Pell JP Fleming M. Association between 5-min Apgar score and attention deficit hyperactivity disorder: a Scotland-wide record linkage study of 758,423 school children. BMC Psychiatry. (2023) 23:794. doi: 10.1186/s12888-023-05217-6 37907891 PMC10619264

[118] Rees S Harding R Walker D. An adverse intrauterine environment: implications for injury and altered development of the brain. Int J Dev Neurosci. (2008) 26:3–11. doi: 10.1016/j.ijdevneu.2007.08.020 17981423

[119] Simon N Volicer L. Neonatal asphyxia in the rat: greater vulnerability of males and persistent effects on brain monoamine synthesis. J Neurochem. (1976) 26:893–900. doi: 10.1111/j.1471-4159.1976.tb06470.x 946815

[120] Hedner T Lundborg P. Catecholamine metabolism in neonatal rat brain during asphyxia and recovery. Acta Physiol Scand. (1980) 109:169–75. doi: 10.1111/j.1748-1716.1980.tb06583.x 6775492

[121] Stott SR Metzakopian E Lin W Kaestner KH Hen R Ang SL. Foxa1 and foxa2 are required for the maintenance of dopaminergic properties in ventral midbrain neurons at late embryonic stages. J Neurosci. (2013) 33:8022–34. doi: 10.1523/JNEUROSCI.4774-12.2013 PMC6618950 23637192

[122] Arcos-Burgos M Castellanos FX Pineda D Lopera F Palacio JD Palacio LG. Attention-deficit/hyperactivity disorder in a population isolate: linkage to loci at 4q13.2, 5q33.3, 11q22, and 17p11. Am J Hum Genet. (2004) 75:998–1014. doi: 10.1086/426154 15497111 PMC1182160

[123] Arcos-Burgos M Jain M Acosta MT Shively S Stanescu H Wallis D. A common variant of the latrophilin 3 gene, LPHN3, confers susceptibility to ADHD and predicts effectiveness of stimulant medication. Mol Psychiatry. (2010) 15:1053–66. doi: 10.1038/mp.2010.6 20157310

[124] Wang S DeLeon C Sun W Quake SR Roth BL Sudhof TC. Alternative splicing of latrophilin-3 controls synapse formation. Nature. (2024) 626:128–35. doi: 10.1038/s41586-023-06913-9 PMC10830413 38233523

[125] Vitobello A Mazel B Lelianova VG Zangrandi A Petitto E Suckling J. ADGRL1 haploinsufficiency causes a variable spectrum of neurodevelopmental disorders in humans and alters synaptic activity and behavior in a mouse model. Am J Hum Genet. (2022) 109:1436–57. doi: 10.1016/j.ajhg.2022.06.011 PMC9388395 35907405

[126] Regan SL Hufgard JR Pitzer EM Sugimoto C Hu YC Williams MT. Knockout of latrophilin-3 in Sprague-Dawley rats causes hyperactivity, hyper-reactivity, under-response to amphetamine, and disrupted dopamine markers. Neurobiol Dis. (2019) 130:104494. doi: 10.1016/j.nbd.2019.104494 31176715 PMC6689430

[127] Yde Ohki CM Grossmann L Alber E Dwivedi T Berger G Werling AM. The stress-Wnt-signaling axis: a hypothesis for attention-deficit hyperactivity disorder and therapy approaches. Transl Psychiatry. (2020) 10:315. doi: 10.1038/s41398-020-00999-9 32948744 PMC7501308

[128] Lin R Learman LN Na CH Renuse S Chen KT Chen PY. Persistently elevated mTOR complex 1-S6 kinase 1 disrupts DARPP-32-dependent D(1) dopamine receptor signaling and behaviors. Biol Psychiatry. (2021) 89:1058–72. doi: 10.1016/j.biopsych.2020.10.012 PMC8076344 33353667

[129] Santiago NA He B Howard SL Beaudin S Strupp BJ Smith DR. Developmental manganese exposure causes lasting attention deficits accompanied by dysregulation of mTOR signaling and catecholaminergic gene expression in brain prefrontal cortex. bioRxiv. (2023). doi: 10.1101/2023.07.16.549215

[130] White R Gatzke-Kopp LM Ryan PJ Lydon-Staley DM. The association between perinatal hypoxia exposure and externalizing symptoms and children's decision making in conditions of uncertainty is moderated by DRD2 genotype. Dev Psychobiol. (2019) 61:56–68. doi: 10.1002/dev.21785 30264459 PMC6318051

[131] Oddsson A Sulem P Sveinbjornsson G Arnadottir GA Steinthorsdottir V Halldorsson GH. Deficit of homozygosity among 1.52 million individuals and genetic causes of recessive lethality. Nat Commun. (2023) 14:3453. doi: 10.1038/s41467-023-38951-2 37301908 PMC10257723

[132] Haavik J Blau N Thony B. Mutations in human monoamine-related neurotransmitter pathway genes. Hum Mutat. (2008) 29:891–902. doi: 10.1002/humu.20700 18444257

[133] Mak CM Lee HC Chan AY Lam CW. Inborn errors of metabolism and expanded newborn screening: review and update. Crit Rev Clin Lab Sci. (2013) 50:142–62. doi: 10.3109/10408363.2013.847896 24295058

[134] Tabarki B Ortigoza-Escobar JD Lee WT AlFadhel M. Editorial: pediatric neurometabolic disorders. Front Neurol. (2021) 12:737398. doi: 10.3389/fneur.2021.737398 34557156 PMC8452847

[135] Spaull RVV Kurian MA. SLC6A3-related dopamine transporter deficiency syndrome. In: Adam MP Feldman J Mirzaa GM Pagon RA Wallace SE Bean LJH, editors. GeneReviews((R)). Seattle (WA: University of Washington (1993). 28749637

[136] Pearson TS Gilbert L Opladen T Garcia-Cazorla A Mastrangelo M Leuzzi V. AADC deficiency from infancy to adulthood: Symptoms and developmental outcome in an international cohort of 63 patients. J Inherit Metab Dis. (2020) 43:1121–30. doi: 10.1002/jimd.12247 PMC7540529 32369189

[137] Wassenberg T Deinum J van Ittersum FJ Kamsteeg EJ Pennings M Verbeek MM. Clinical presentation and long-term follow-up of dopamine beta hydroxylase deficiency. J Inherit Metab Dis. (2021) 44:554–65. doi: 10.1002/jimd.12321 PMC8246878 33034372

[138] Heyne HO Karjalainen J Karczewski KJ Lemmela SM Zhou W FinnGen. Mono- and biallelic variant effects on disease at biobank scale. Nature. (2023) 613:519–25. doi: 10.1038/s41586-022-05420-7 PMC9849130 36653560

[139] Cannon Homaei S Barone H Kleppe R Betari N Reif A Haavik J. ADHD symptoms in neurometabolic diseases: Underlying mechanisms and clinical implications. Neurosci Biobehav Rev. (2022) 132:838–56. doi: 10.1016/j.neubiorev.2021.11.012 34774900

[140] Barone H Bliksrud YT Elgen IB Szigetvari PD Kleppe R Ghorbani S. Tyrosinemia Type 1 and symptoms of ADHD: Biochemical mechanisms and implications for treatment and prognosis. Am J Med Genet B Neuropsychiatr Genet. (2020) 183:95–105. doi: 10.1002/ajmg.b.32764 31633311

[141] Stevenson M McNaughton N. A comparison of phenylketonuria with attention deficit hyperactivity disorder: do markedly different aetiologies deliver common phenotypes? Brain Res Bull. (2013) 99:63–83. doi: 10.1016/j.brainresbull.2013.10.003 24140048

[142] Barone H Elgen IB Bliksrud YT Vangsoy Hansen E Skavhellen RR Furevik MI. Case report: ADHD and prognosis in tyrosinemia type 1. Front Psychiatry. (2023) 14:1213590. doi: 10.3389/fpsyt.2023.1213590 37533886 PMC10392124

[143] Thimm E Herebian D Assmann B Klee D Mayatepek E Spiekerkoetter U. Increase of CSF tyrosine and impaired serotonin turnover in tyrosinemia type I. Mol Genet Metab. (2011) 102:122–5. doi: 10.1016/j.ymgme.2010.11.003 21112803

[144] Segawa M Hosaka A Miyagawa F Nomura Y Imai H. Hereditary progressive dystonia with marked diurnal fluctuation. Adv Neurol. (1976) 14:215–33. 945938

[145] Weissbach A Pauly MG Herzog R Hahn L Halmans S Hamami F. Relationship of genotype, phenotype, and treatment in dopa-responsive dystonia: MDSGene review. Mov Disord. (2022) 37:237–52. doi: 10.1002/mds.28874 34908184

[146] Percudani R Peracchi A. A genomic overview of pyridoxal-phosphate-dependent enzymes. EMBO Rep. (2003) 4:850–4. doi: 10.1038/sj.embor.embor914 PMC1326353 12949584

[147] Stach K Stach W Augoff K. Vitamin B6 in health and disease. Nutrients. (2021) 13. doi: 10.3390/nu13093229 PMC8467949 34579110

[148] Kinlin LM Weinstein M. Scurvy: old disease, new lessons. Paediatr Int Child Health. (2023) 43:83–94. doi: 10.1080/20469047.2023.2262787 37795755

[149] Ernst M Zametkin AJ Matochik JA Pascualvaca D Jons PH Cohen RM. High midbrain [18F]DOPA accumulation in children with attention deficit hyperactivity disorder. Am J Psychiatry. (1999) 156:1209–15. doi: 10.1176/ajp.156.8.1209 10450262

[150] Torstenson R Hartvig P Langstrom B Westerberg G Tedroff J. Differential effects of levodopa on dopaminergic function in early and advanced Parkinson's disease. Ann Neurol. (1997) 41:334–40. doi: 10.1002/ana.410410308 9066354

[151] Abercrombie ED Bonatz AE Zigmond MJ. Effects of L-dopa on extracellular dopamine in striatum of normal and 6-hydroxydopamine-treated rats. Brain Res. (1990) 525:36–44. doi: 10.1016/0006-8993(90)91318-B 2123121

[152] Wolf ME Roth RH. Autoreceptor regulation of dopamine synthesis. Ann N Y Acad Sci. (1990) 604:323–43. doi: 10.1111/j.1749-6632.1990.tb32003.x 2171398

[153] Ludolph AG Kassubek J Schmeck K Glaser C Wunderlich A Buck AK. Dopaminergic dysfunction in attention deficit hyperactivity disorder (ADHD), differences between pharmacologically treated and never treated young adults: a 3,4-dihdroxy-6-[18F]fluorophenyl-l-alanine PET study. Neuroimage. (2008) 41:718–27. doi: 10.1016/j.neuroimage.2008.02.025 18424180

[154] Spencer TJ Biederman J Madras BK Dougherty DD Bonab AA Livni E. Further evidence of dopamine transporter dysregulation in ADHD: a controlled PET imaging study using altropane. Biol Psychiatry. (2007) 62:1059–61. doi: 10.1016/j.biopsych.2006.12.008 PMC2715944 17511972

[155] Cheon KA Ryu YH Kim YK Namkoong K Kim CH Lee JD. Dopamine transporter density in the basal ganglia assessed with [123I]IPT SPET in children with attention deficit hyperactivity disorder. Eur J Nucl Med Mol Imaging. (2003) 30:306–11. doi: 10.1007/s00259-002-1047-3 12552351

[156] Dougherty DD Bonab AA Spencer TJ Rauch SL Madras BK Fischman AJ. Dopamine transporter density in patients with attention deficit hyperactivity disorder. Lancet. (1999) 354:2132–3. doi: 10.1016/S0140-6736(99)04030-1 10609822

[157] Dresel S Krause J Krause KH LaFougere C Brinkbaumer K Kung HF. Attention deficit hyperactivity disorder: binding of [99mTc]TRODAT-1 to the dopamine transporter before and after methylphenidate treatment. Eur J Nucl Med. (2000) 27:1518–24. doi: 10.1007/s002590000330 11083541

[158] Jucaite A Fernell E Halldin C Forssberg H Farde L. Reduced midbrain dopamine transporter binding in male adolescents with attention-deficit/hyperactivity disorder: association between striatal dopamine markers and motor hyperactivity. Biol Psychiatry. (2005) 57:229–38. doi: 10.1016/j.biopsych.2004.11.009 15691523

[159] van Dyck CH Quinlan DM Cretella LM Staley JK Malison RT Baldwin RM. Unaltered dopamine transporter availability in adult attention deficit hyperactivity disorder. Am J Psychiatry. (2002) 159:309–12. doi: 10.1176/appi.ajp.159.2.309 11823278

[160] del Campo N Fryer TD Hong YT Smith R Brichard L Acosta-Cabronero J. A positron emission tomography study of nigro-striatal dopaminergic mechanisms underlying attention: implications for ADHD and its treatment. Brain. (2013) 136:3252–70. doi: 10.1093/brain/awt263 PMC4125626 24163364

[161] Wiers CE Lohoff FW Lee J Muench C Freeman C Zehra A. Methylation of the dopamine transporter gene in blood is associated with striatal dopamine transporter availability in ADHD: A preliminary study. Eur J Neurosci. (2018) 48:1884–95. doi: 10.1111/ejn.2018.48.issue-3 PMC6113083 30033547

[162] Ernst M Zametkin AJ Matochik JA Jons PH Cohen RM. DOPA decarboxylase activity in attention deficit hyperactivity disorder adults. A [fluorine-18]fluorodopa positron emission tomographic study. J Neurosci. (1998) 18:5901–7. doi: 10.1523/JNEUROSCI.18-15-05901.1998 PMC6793062 9671677

[163] Volkow ND Wang GJ Newcorn J Telang F Solanto MV Fowler JS. Depressed dopamine activity in caudate and preliminary evidence of limbic involvement in adults with attention-deficit/hyperactivity disorder. Arch Gen Psychiatry. (2007) 64:932–40. doi: 10.1001/archpsyc.64.8.932 17679638

[164] Volkow ND Wang GJ Newcorn J Fowler JS Telang F Solanto MV. Brain dopamine transporter levels in treatment and drug naive adults with ADHD. Neuroimage. (2007) 34:1182–90. doi: 10.1016/j.neuroimage.2006.10.014 17126039

[165] Forssberg H Fernell E Waters S Waters N Tedroff J. Altered pattern of brain dopamine synthesis in male adolescents with attention deficit hyperactivity disorder. Behav Brain Funct. (2006) 2:40. doi: 10.1186/1744-9081-2-40 17144907 PMC1698925

[166] Nikolaus S Mamlins E Giesel FL Schmitt D Muller HW. Monoaminergic hypo- or hyperfunction in adolescent and adult attention-deficit hyperactivity disorder? Rev Neurosci. (2022) 33:347–64. doi: 10.1515/revneuro-2021-0083 34378877

[167] Badgaiyan RD Sinha S Sajjad M Wack DS. Attenuated tonic and enhanced phasic release of dopamine in attention deficit hyperactivity disorder. PLoS One. (2015) 10:e0137326. doi: 10.1371/journal.pone.0137326 26422146 PMC4589406

[168] Yamamoto M Inada T. Positron emission tomography studies in adult patients with attention-deficit/hyperactivity disorder. Jpn J Radiol. (2023) 41:382–92. doi: 10.1007/s11604-022-01368-w 36480104

[169] Millevert C Vidas-Guscic N Vanherp L Jonckers E Verhoye M Staelens S. Resting-state functional MRI and PET imaging as noninvasive tools to study (Ab)Normal neurodevelopment in humans and rodents. J Neurosci. (2023) 43:8275–93. doi: 10.1523/JNEUROSCI.1043-23.2023 PMC10711730 38073598

[170] Gainetdinov RR Wetsel WC Jones SR Levin ED Jaber M Caron MG. Role of serotonin in the paradoxical calming effect of psychostimulants on hyperactivity. Science. (1999) 283:397–401. doi: 10.1126/science.283.5400.397 9888856

[171] Bekar LK Wei HS Nedergaard M. The locus coeruleus-norepinephrine network optimizes coupling of cerebral blood volume with oxygen demand. J Cereb Blood Flow Metab. (2012) 32:2135–45. doi: 10.1038/jcbfm.2012.115 PMC3519408 22872230

[172] Renden RB Institoris A Sharma K Tran CHT. Modulatory effects of noradrenergic and serotonergic signaling pathway on neurovascular coupling. Commun Biol. (2024) 7:287. doi: 10.1038/s42003-024-05996-y 38459113 PMC10923894

[173] Su S Zhao J Dai Y Lin L Zhou Q Yan Z. Altered neurovascular coupling in the children with attention-deficit/hyperactivity disorder: a comprehensive fMRI analysis. Eur Child Adolesc Psychiatry. (2024) 33:1081–91. doi: 10.1007/s00787-023-02238-0 37222790

[174] Chen Y Wang M Su S Dai Y Zou M Lin L. Assessment of the glymphatic function in children with attention-deficit/hyperactivity disorder. Eur Radiol. (2024) 34:1444–52. doi: 10.1007/s00330-023-10220-2 37673963

[175] Braak H Ghebremedhin E Rub U Bratzke H Del Tredici K. Stages in the development of Parkinson's disease-related pathology. Cell Tissue Res. (2004) 318:121–34. doi: 10.1007/s00441-004-0956-9 15338272

[176] Ballanger B van Eimeren T Moro E Lozano AM Hamani C Boulinguez P. Stimulation of the subthalamic nucleus and impulsivity: release your horses. Ann Neurol. (2009) 66:817–24. doi: 10.1002/ana.21795 PMC2972250 20035509

[177] Gerfen CR Engber TM Mahan LC Susel Z Chase TN Monsma FJ Jr.. D1 and D2 dopamine receptor-regulated gene expression of striatonigral and striatopallidal neurons. Science. (1990) 250:1429–32. doi: 10.1126/science.2147780 2147780

[178] Obeso JA Guridi J Nambu A Crossman AR. Motor manifestations and basal ganglia output activity: the paradox continues. Mov Disord. (2013) 28:416–8. doi: 10.1002/mds.25358 23494928

[179] Alexander GE Crutcher MD. Functional architecture of basal ganglia circuits: neural substrates of parallel processing. Trends Neurosci. (1990) 13:266–71. doi: 10.1016/0166-2236(90)90107-L 1695401

[180] Albin RL Young AB Penney JB. The functional anatomy of basal ganglia disorders. Trends Neurosci. (1989) 12:366–75. doi: 10.1016/0166-2236(89)90074-X 2479133

[181] Fliers E Rommelse N Vermeulen SH Altink M Buschgens CJ Faraone SV. Motor coordination problems in children and adolescents with ADHD rated by parents and teachers: effects of age and gender. J Neural Transm (Vienna). (2008) 115:211–20. doi: 10.1007/s00702-007-0827-0 17994185

[182] Demers MM McNevin N Azar NR. ADHD and motor control: A review of the motor control deficiencies associated with attention deficit/hyperactivity disorder and current treatment options. Crit Reviews™ Phys Rehabil Med. (2013) 25:231–9. doi: 10.1615/CritRevPhysRehabilMed.2013009763

[183] Kaiser ML Schoemaker MM Albaret JM Geuze RH. What is the evidence of impaired motor skills and motor control among children with attention deficit hyperactivity disorder (ADHD)? Systematic review of the literature. Res Dev Disabil. (2015) 36C:338–57. doi: 10.1016/j.ridd.2014.09.023 25462494

[184] Rubia K Taylor E Smith AB Oksanen H Overmeyer S Newman S. Neuropsychological analyses of impulsiveness in childhood hyperactivity. Br J Psychiatry. (2001) 179:138–43. doi: 10.1192/bjp.179.2.138 11483475

[185] Smith AB Taylor E Brammer M Toone B Rubia K. Task-specific hypoactivation in prefrontal and temporoparietal brain regions during motor inhibition and task switching in medication-naive children and adolescents with attention deficit hyperactivity disorder. Am J Psychiatry. (2006) 163:1044–51. doi: 10.1176/ajp.2006.163.6.1044 16741205

[186] Sagvolden T Johansen EB Aase H Russell VA. A dynamic developmental theory of attention-deficit/hyperactivity disorder (ADHD) predominantly hyperactive/impulsive and combined subtypes. Behav Brain Sci. (2005) 28:397–419. doi: 10.1017/S0140525X05000075 16209748

[187] Kalff AC de Sonneville LM Hurks PP Hendriksen JG Kroes M Feron FJ. Low- and high-level controlled processing in executive motor control tasks in 5-6-year-old children at risk of ADHD. J Child Psychol Psychiatry. (2003) 44:1049–57. doi: 10.1111/jcpp.2003.44.issue-7 14531587

[188] Meyer A Sagvolden T. Fine motor skills in South African children with symptoms of ADHD: influence of subtype, gender, age, and hand dominance. Behav Brain Funct. (2006) 2:33. doi: 10.1186/1744-9081-2-33 17029638 PMC1626473

[189] Berquin PC Giedd JN Jacobsen LK Hamburger SD Krain AL Rapoport JL. Cerebellum in attention-deficit hyperactivity disorder: a morphometric MRI study. Neurology. (1998) 50:1087–93. doi: 10.1212/WNL.50.4.1087 9566399

[190] Mulder MJ Baeyens D Davidson MC Casey BJ EV DENB VANE H. Familial vulnerability to ADHD affects activity in the cerebellum in addition to the prefrontal systems. J Am Acad Child Adolesc Psychiatry. (2008) 47:68–75. doi: 10.1097/chi.0b013e31815a56dc 18174827

[191] Perlov E Tebarzt van Elst L Buechert M Maier S Matthies S Ebert D. H(1)-MR-spectroscopy of cerebellum in adult attention deficit/hyperactivity disorder. J Psychiatr Res. (2010) 44:938–43. doi: 10.1016/j.jpsychires.2010.02.016 20332052

[192] Pitcher TM Piek JP Hay DA. Fine and gross motor ability in males with ADHD. Dev Med Child Neurol. (2003) 45:525–35. doi: 10.1111/j.1469-8749.2003.tb00952.x 12882531

[193] Hoshi E Tremblay L Feger J Carras PL Strick PL. The cerebellum communicates with the basal ganglia. Nat Neurosci. (2005) 8:1491–3. doi: 10.1038/nn1544 16205719

[194] Koziol LF Budding D Andreasen N D'Arrigo S Bulgheroni S Imamizu H. Consensus paper: the cerebellum's role in movement and cognition. Cerebellum. (2014) 13:151–77. doi: 10.1007/s12311-013-0511-x PMC4089997 23996631

[195] Sawamoto N Piccini P Hotton G Pavese N Thielemans K Brooks DJ. Cognitive deficits and striato-frontal dopamine release in Parkinson's disease. Brain. (2008) 131:1294–302. doi: 10.1093/brain/awn054 18362097

[196] Vaillancourt DE Schonfeld D Kwak Y Bohnen NI Seidler R. Dopamine overdose hypothesis: evidence and clinical implications. Mov Disord. (2013) 28:1920–9. doi: 10.1002/mds.v28.14 PMC3859825 24123087

[197] Cools R. Dopaminergic modulation of cognitive function-implications for L-DOPA treatment in Parkinson's disease. Neurosci Biobehav Rev. (2006) 30:1–23. doi: 10.1016/j.neubiorev.2005.03.024 15935475

[198] Heilman KM Voeller KK Nadeau SE. A possible pathophysiologic substrate of attention deficit hyperactivity disorder. J Child Neurol. (1991) 6 Suppl:S76–81. doi: 10.1177/0883073891006001S09 2002218

[199] Oades RD. Dopamine-serotonin interactions in attention-deficit hyperactivity disorder (ADHD). Prog Brain Res. (2008) 172:543–65. doi: 10.1016/S0079-6123(08)00926-6 18772050

[200] Rommelse NN Altink ME de Sonneville LM Buschgens CJ Buitelaar J Oosterlaan J. Are motor inhibition and cognitive flexibility dead ends in ADHD? J Abnorm Child Psychol. (2007) 35:957–67. doi: 10.1007/s10802-007-9146-z 17503173

[201] Karalunas SL Huang-Pollock CL. Integrating impairments in reaction time and executive function using a diffusion model framework. J Abnorm Child Psychol. (2013) 41:837–50. doi: 10.1007/s10802-013-9715-2 PMC3679296 23334775

[202] Salum GA Sonuga-Barke E Sergeant J Vandekerckhove J Gadelha A Moriyama TS. Mechanisms underpinning inattention and hyperactivity: neurocognitive support for ADHD dimensionality. Psychol Med. (2014) 44:3189–201. doi: 10.1017/S0033291714000919 25065454

[203] Kaasinen V Nurmi E Bruck A Eskola O Bergman J Solin O. Increased frontal [(18)F]fluorodopa uptake in early Parkinson's disease: sex differences in the prefrontal cortex. Brain. (2001) 124:1125–30. doi: 10.1093/brain/124.6.1125 11353728

[204] Rakshi JS Uema T Ito K Bailey DL Morrish PK Ashburner J. Frontal, midbrain and striatal dopaminergic function in early and advanced Parkinson's disease A 3D [(18)F]dopa-PET study. Brain. (1999) 122:1637–50. doi: 10.1093/brain/122.9.1637 10468504

[205] Zigmond MJ Abercrombie ED Berger TW Grace AA Stricker EM. Compensations after lesions of central dopaminergic neurons: some clinical and basic implications. Trends Neurosci. (1990) 13:290–6. doi: 10.1016/0166-2236(90)90112-N 1695406

[206] Jahanshahi M Jones CR Zijlmans J Katzenschlager R Lee L Quinn N. Dopaminergic modulation of striato-frontal connectivity during motor timing in Parkinson's disease. Brain. (2010) 133:727–45. doi: 10.1093/brain/awq012 20305278

[207] Kolachana BS Saunders RC Weinberger DR. Augmentation of prefrontal cortical monoaminergic activity inhibits dopamine release in the caudate nucleus: an in *vivo* neurochemical assessment in the rhesus monkey. Neuroscience. (1995) 69:859–68. doi: 10.1016/0306-4522(95)00246-F 8596654

[208] Roberts AC De Salvia MA Wilkinson LS Collins P Muir JL Everitt BJ. 6-Hydroxydopamine lesions of the prefrontal cortex in monkeys enhance performance on an analog of the Wisconsin Card Sort Test: possible interactions with subcortical dopamine. J Neurosci. (1994) 14:2531–44. doi: 10.1523/JNEUROSCI.14-05-02531.1994 PMC6577476 8182426

[209] Weintraub D Siderowf AD Potenza MN Goveas J Morales KH Duda JE. Association of dopamine agonist use with impulse control disorders in Parkinson disease. Arch Neurol. (2006) 63:969–73. doi: 10.1001/archneur.63.7.969 PMC1761054 16831966

[210] Voon V Potenza MN Thomsen T. Medication-related impulse control and repetitive behaviors in Parkinson's disease. Curr Opin Neurol. (2007) 20:484–92. doi: 10.1097/WCO.0b013e32826fbc8f 17620886

[211] Russell VA. Hypodopaminergic and hypernoradrenergic activity in prefrontal cortex slices of an animal model for attention-deficit hyperactivity disorder–the spontaneously hypertensive rat. Behav Brain Res. (2002) 130:191–6. doi: 10.1016/S0166-4328(01)00425-9 11864734

[212] Norman LJ Carlisi C Lukito S Hart H Mataix-Cols D Radua J. Structural and functional brain abnormalities in attention-deficit/hyperactivity disorder and obsessive-compulsive disorder: A comparative meta-analysis. JAMA Psychiatry. (2016) 73:815–25. doi: 10.1001/jamapsychiatry.2016.0700 27276220

[213] Lukito S Norman L Carlisi C Radua J Hart H Simonoff E. Comparative meta-analyses of brain structural and functional abnormalities during cognitive control in attention-deficit/hyperactivity disorder and autism spectrum disorder. Psychol Med. (2020) 50:894–919. doi: 10.1017/S0033291720000574 32216846 PMC7212063

[214] Hart H Radua J Nakao T Mataix-Cols D Rubia K. Meta-analysis of functional magnetic resonance imaging studies of inhibition and attention in attention-deficit/hyperactivity disorder: exploring task-specific, stimulant medication, and age effects. JAMA Psychiatry. (2013) 70:185–98. doi: 10.1001/jamapsychiatry.2013.277 23247506

[215] Goldman-Rakic PS Muly EC 3rd Williams GV. D(1) receptors in prefrontal cells and circuits. Brain Res Brain Res Rev. (2000) 31:295–301. doi: 10.1016/S0165-0173(99)00045-4 10719156

[216] Congdon E Constable RT Lesch KP Canli T. Influence of SLC6A3 and COMT variation on neural activation during response inhibition. Biol Psychol. (2009) 81:144–52. doi: 10.1016/j.biopsycho.2009.03.005 PMC2689843 19482231

[217] Farrell SM Tunbridge EM Braeutigam S Harrison PJ. COMT Val(158)Met genotype determines the direction of cognitive effects produced by catechol-O-methyltransferase inhibition. Biol Psychiatry. (2012) 71:538–44. doi: 10.1016/j.biopsych.2011.12.023 PMC3314969 22364739

[218] Brennan AR Arnsten AF. Neuronal mechanisms underlying attention deficit hyperactivity disorder: the influence of arousal on prefrontal cortical function. Ann N Y Acad Sci. (2008) 1129:236–45. doi: 10.1196/nyas.2008.1129.issue-1 PMC2863119 18591484

[219] MacDonald HJ Stinear CM Ren A Coxon JP Kao J Macdonald L. Dopamine gene profiling to predict impulse control and effects of dopamine agonist ropinirole. J Cognit Neurosci. (2016) 28:909–19. doi: 10.1162/jocn_a_00946 26942320

[220] Hegvik TA Jacobsen KK Fredriksen M Zayats T Haavik J. A candidate gene investigation of methylphenidate response in adult attention-deficit/hyperactivity disorder patients: results from a naturalistic study. J Neural Transm (Vienna). (2016) 123:859–65. doi: 10.1007/s00702-016-1540-7 PMC4969350 27091191

[221] Haavik J. Genome guided personalized drug therapy in attention deficit hyperactivity disorder. Front Psychiatry. (2022) 13:925442. doi: 10.3389/fpsyt.2022.925442 35832601 PMC9271625

[222] Ferranti AS Luessen DJ Niswender CM. Novel pharmacological targets for GABAergic dysfunction in ADHD. Neuropharmacology. (2024) 249:109897. doi: 10.1016/j.neuropharm.2024.109897 38462041 PMC11843668

[223] Pagan AF Huizar YP Short TR Gotcher Z Schmidt AT. Adult attention-deficit/hyperactivity disorder: a narrative review of biological mechanisms, treatments, and outcomes. Curr Neurol Neurosci Rep. (2023) 23:451–60. doi: 10.1007/s11910-023-01280-4 37335460

[224] Faraone SV Banaschewski T Coghill D Zheng Y Biederman J Bellgrove MA. The World Federation of ADHD International Consensus Statement: 208 Evidence-based conclusions about the disorder. Neurosci Biobehav Rev. (2021) 128:789–818. doi: 10.1016/j.neubiorev.2021.01.022 33549739 PMC8328933

[225] Mawlawi O Pan T Macapinlac HA. PET/CT imaging techniques, considerations, and artifacts. J Thorac Imaging. (2006) 21:99–110. doi: 10.1097/00005382-200605000-00002 16770227

[226] Veronneau-Veilleux F Robaey P Ursino M Nekka F. A mechanistic model of ADHD as resulting from dopamine phasic/tonic imbalance during reinforcement learning. Front Comput Neurosci. (2022) 16:849323. doi: 10.3389/fncom.2022.849323 35923915 PMC9342605

[227] Gkougka D Mitropoulos K Tzanakaki G Panagouli E Psaltopoulou T Thomaidis L. Gut microbiome and attention deficit/hyperactivity disorder: a systematic review. Pediatr Res. (2022) 92:1507–19. doi: 10.1038/s41390-022-02027-6 35354932

